# Phylogeny of Parasitic Parabasalia and Free-Living Relatives Inferred from Conventional Markers *vs. Rpb1*, a Single-Copy Gene

**DOI:** 10.1371/journal.pone.0020774

**Published:** 2011-06-09

**Authors:** Shehre-Banoo Malik, Cynthia D. Brochu, Ivana Bilic, Jing Yuan, Michael Hess, John M. Logsdon, Jane M. Carlton

**Affiliations:** 1 Department of Microbiology, Division of Medical Parasitology, New York University School of Medicine, New York, New York, United States of America; 2 Department of Biology, Roy J. Carver Center for Comparative Genomics, University of Iowa, Iowa City, Iowa, United States of America; 3 Department for Farm Animals and Veterinary Public Health, Clinic for Avian, Reptile and Fish Medicine, University of Veterinary Medicine, Vienna, Austria; Université Paris Sud, France

## Abstract

**Background:**

Parabasalia are single-celled eukaryotes (protists) that are mainly comprised of endosymbionts of termites and wood roaches, intestinal commensals, human or veterinary parasites, and free-living species. Phylogenetic comparisons of parabasalids are typically based upon morphological characters and 18S ribosomal RNA gene sequence data (rDNA), while biochemical or molecular studies of parabasalids are limited to a few axenically cultivable parasites. These previous analyses and other studies based on PCR amplification of duplicated protein-coding genes are unable to fully resolve the evolutionary relationships of parabasalids. As a result, genetic studies of Parabasalia lag behind other organisms.

**Principal Findings:**

Comparing parabasalid EF1α, α-tubulin, enolase and MDH protein-coding genes with information from the *Trichomonas vaginalis* genome reveals difficulty in resolving the history of species or isolates apart from duplicated genes. A conserved single-copy gene encodes the largest subunit of RNA polymerase II (Rpb1) in *T. vaginalis* and other eukaryotes. Here we directly sequenced *Rpb1* degenerate PCR products from 10 parabasalid genera, including several *T. vaginalis* isolates and avian isolates, and compared these data by phylogenetic analyses. *Rpb1* genes from parabasalids, diplomonads, *Parabodo*, *Diplonema* and *Percolomonas* were all intronless, unlike intron-rich homologs in *Naegleria*, *Jakoba* and *Malawimonas*.

**Conclusions/Significance:**

The phylogeny of Rpb1 from parasitic and free-living parabasalids, and conserved Rpb1 insertions, support Trichomonadea, Tritrichomonadea, and Hypotrichomonadea as monophyletic groups. These results are consistent with prior analyses of rDNA and GAPDH sequences and ultrastructural data. The Rpb1 phylogenetic tree also resolves species- and isolate-level relationships. These findings, together with the relative ease of Rpb1 isolation, make it an attractive tool for evaluating more extensive relationships within Parabasalia.

## Introduction

Parabasalia belongs to the supergroup Excavata, subgroup Metamonada [1], [2], [3], [4], [5], [6], and consists of single-celled flagellated eukaryotes that include parasites and commensals of vertebrate hosts, commensals and endosymbionts of invertebrates, and a few described free-living species. Among the parasitic parabasalids, several are important agents of human urogenital, subgingival, oral, bronchial and gastrointestinal infections. Historically, Parabasalia were divided into two groups based upon morphological characters observed mainly by light microscopy. Large (∼200 µm) multiflagellated forms typically found in termite and cockroach hindguts are commonly referred to as “hypermastigotes” and smaller (∼10–20 µm) flagellates, found in both vertebrate and invertebrate hosts, are called “trichomonads”. However, recent morphological and molecular phylogenetic analyses recover six parabasalid groups [7], [8], [9], [10], [11]: Trichomonadea, Tritrichomonadea, Hypotrichomonadea, Cristamonadea, Spirotrichonymphea and Trichonymphea. The relationships within and among the six groups are not fully resolved [11].

Genetic markers are powerful tools for rapid identification of parasites and other microbes from patient specimens and environmental samples. Studies in Parabasalia have fallen behind those in other organisms partly due to problems with obtaining robust genetic markers. There is a pressing need for more informative genetic markers in the Parabasalia and their relatives in the supergroup Excavata, since a stronger phylogenetic framework would improve our understanding of the biology of the diverse species found within this group. An improved parabasalid phylogeny will also help advance comparative genomics within this group, and serve as a guide in the choice of which parabasalids to target for genome sequencing. Here we present a critical examination of recent molecular phylogenetic analyses of Parabasalia and implement a new molecular phylogenetic marker for resolving the evolutionary relationships within Parabasalia and its relatives.

Parabasalids are interesting since they include species of medical and veterinary importance, and ecologically relevant models of host-symbiont coevolution. Parabasalia is a highly diverged lineage of eukaryotic microorganisms [5], [12] whose members exhibit unusual definitive metabolic and cytoskeletal properties such as the presence of hydrogenosomes (derived from mitochondria), and a parabasal apparatus consisting of the Golgi body attached to striated fibers near the karyomastigont (a structure comprised of a nucleus and four basal bodies, that anchor the three anterior and one recurrent flagellum). In this group, the Trichomonadea, Tritrichomonadea and Hypotrichomonadea are of primary concern to parasitologists; however, their evolutionary relationships are not well resolved. *Trichomonas vaginalis* causes the most prevalent non-viral sexually transmitted infection in humans worldwide, and a draft genome was recently published [13], [14]. *Trichomonas tenax* infects the human oral cavity, usually in the subgingival space [15]. Cases of human respiratory and pulmonary infections involving *T. tenax*, *T. vaginalis*, *Tetratrichomonas* sp. and *Pentatrichomonas hominis* have been reported [16], [17], [18], [19], [20]. *Dientamoeba fragilis* causes human gastrointestinal disease [21], [22]. In addition, *Tetratrichomonas gallinarum* and *Trichomonas gallinae* are found in the digestive tract of birds [23], [24], [25]. *Trichomonas gallinae* is an etiological agent of avian trichomonosis, a disease especially affecting pigeons and raptors [26], [27], while *Tetratrichomonas gallinarum* is disputed as a primary pathogen [28], [29] as it is often found together with another parabasalid, *Histomonas meleagridis*, in the caecum and liver of naturally infected chickens and turkeys [30], [31]. *Tritrichomonas foetus* causes sexually transmitted infections in cattle that result in spontaneous abortion. *T. foetus* and *P. hominis* also cause diarrhea in domestic cats and dogs [32], [33], [34], [35]. *Monocercomonas* and *Trichomitus* have a broad host range including amphibians, reptiles, mammals and arthropods, and *Hypotrichomonas acosta* is found in the gastrointestinal tracts of snakes and several lizard species [36]. Most other reported non-parasitic parabasalids live in the hindguts of termites or cockroaches, except for a few free-living species such as *Pseudotrichomonas keilini* and *Monotrichomonas carabina* [11], [37].

**Figure 1:**
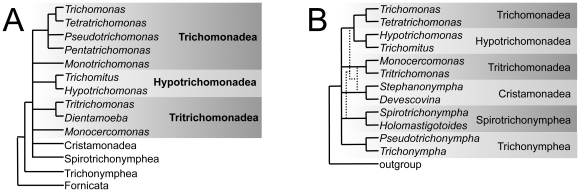
Cartoon of parabasalid evolutionary relationships summarized from published phylogenies. The consensus backbone phylogenies shown are derived from (**A**) 18S rDNA [10], [37], and (**B**) concatenated 18S rDNA genes and enolase, GAPDH, α- and β-tubulin proteins [11]. Dotted lines indicate prior results without 18S rDNA [7].

Evolutionary relationships of parabasalids are under constant revision [8], [10], [11], [38]. Historically, genes encoding the 18S and 5.8S ribosomal RNA subunits (rDNA) have been used to infer parabasalid relationships at the greatest taxonomic breadth [39], [40], [41], but many parts of these molecular phylogenies are unresolved [9], [11], [42]. A cartoon consensus of recent 18S rDNA phylogenies of a number of parabasalids is summarized in **Figure 1**. Cloned degenerate polymerase chain reaction (PCR) products of several genes encoding proteins such as glyceraldehyde 3-phosphate dehydrogenase (GAPDH) [8], [9], [43], malate dehydrogenase (MDH) [44], enolase [45], α- and β-tubulin [7], [11], [46] have also been used to infer the evolutionary relationships, albeit of a less taxonomically-broad representation of the six parabasalid groups. However, these markers are not ideal: parabasalid enolase genes exhibit recombination [45], and *MDH* and *GAPDH* genes appear to be most closely related to bacterial homologs *via* lateral gene transfer [43], [44], [47], [48]. In contrast, α- and β-tubulin genes are more similar to eukaryotic homologs [7], [46], making these two and rDNA the only genes available until now for comparison of parabasalids to other eukaryotes. However, all of these protein-coding genes can be found duplicated in various parabasalid genera, and individually lack resolution at different taxonomic levels, while their phylogenies do not strongly corroborate one another [7], [43], [46], [49]. In spite of this, both *GAPDH* and 18S rRNA genes typically converge upon the same six monophyletic groups [8], [9] and thus probably contribute most of the signals to published analyses of concatenated parabasalid genes. These data suggest that an alternate eukaryotic protein-coding gene that has not undergone recombination, horizontal gene transfer, or duplication might be more useful to resolve the relationships within Parabasalia and between parabasalids and other eukaryotes.

Parabasalids tend to exhibit large genome sizes in contrast with other parasites [50], consistent with widespread gene duplication and the presence of families of transposable elements, as revealed in the ∼160 Mb genome sequence of *T. vaginalis* [13], [51], [52], [53]. The widespread presence of duplicated genes makes it more difficult to select phylogenetically informative protein-coding genes for comparison at the same taxonomic breadth as rDNA markers in this phylum, and further restricts our ability to resolve relationships between species and conspecific isolates of parabasalids. The highly repetitive nature of genomes in this group, together with an inability to establish pure cultures of diverse representative parabasalids make it likely that any taxonomically-broad molecular phylogenetic survey of Parabasalia will continue to rely on using a degenerate PCR technique (rather than whole genome or transcriptome surveys) to gather sequence data from genes chosen to elucidate the species tree. Furthermore, single-copy genes are useful cytogenetic tags for distinguishing chromosomes, and would be useful to eventually establish genetic maps in parabasalids [54], [55]. A protein-coding genetic marker for parabasalids that is easily isolated and unlikely to evolve by gene duplication, horizontal gene transfer or gene loss is needed to: (i) compare and corroborate with the morphological and rDNA molecular phylogeny; (ii) improve our resolution of relationships among and within major groups; and (iii) enable reliable species-level identification of field isolates.

Consequently, the goal of this study was to investigate the evolutionary relationships of a few representative cultivable parasitic parabasalids relative to *T. vaginalis* lab strain G3, in order to test several previous classifications, and evaluate the genetic distance of candidates for further comparative genomic analyses. The relationships of some of these organisms are unclear from analyses of the loci conventionally used to compare diverse parabasalids. While useful markers in many ways, single-copy genes in the *T. vaginalis* genome [13] are not always conserved enough among eukaryotes to be suitable candidates for the design of degenerate primers for PCR (ref. [56] illustrates phylogenies of a few variable but conserved exemplar proteins). Conventionally used multicopy genes may be easier to amplify with apparent high yields by degenerate PCR than single-copy genes, especially from scarce uncultivable specimens, however they may also lack resolution at various taxonomic levels. In Parabasalia these genes exhibit difficulty both in resolving conspecific isolates and in resolving the relationships between the most distantly related members of the group. In this study we examine whether a well-conserved single-copy gene corroborates the phylogenetic relationships of parasitic parabasalids determined by conventional markers, using similar analytical methods.

*Rpb1*, a ubiquitous eukaryotic gene coding for the largest subunit of RNA polymerase II, is a single-copy gene in *T. vaginalis* isolate NIH:C1 as demonstrated by Southern blot analysis [57], a single-copy gene in the complete genome sequence of *T. vaginalis* isolate G3 [13], and also a single-copy gene in most eukaryotes [58]. These characteristics and its large (∼5 kb) intronless state in *T. vaginalis* [57], indicate potential utility of *Rpb1* sequence data for inferring the phylogeny of groups within Parabasalia. *Pms1*, a *mutL* homolog, is another potentially useful (and likely single-copy) genetic marker in *T. vaginalis* that is ubiquitous in other eukaryotes [54], [56]. Here we report revised phylogenetic analyses of new and existing parabasalid data from conventionally used protein-coding genes, and the first phylogeny of Rpb1 proteins from a few parasitic and free-living parabasalids and related microorganisms in the Excavata. We encourage other investigators to begin using single-copy *Rpb1* and *Pms1* genes to improve the phylogenetic resolution of additional parabasalids and their relatives, following this study.

## Results and Discussion

Recent analyses of 18S rDNA [10], GAPDH [8], [9], and concatenated α- and β-tubulin, enolase, glyceraldehyde-3-phosphate dehydrogenase (GAPDH) protein and 18S rDNA [7], [11], [49] sequence data converge on dividing Parabasalia into six groups (**Figure 1**). Membership within, and the relationships between, these six groups are ambiguous, depending on the taxon sampling and chosen outgroup; for example, *Monotrichomonas* is not always resolved as a member of the Trichomonadea, and the position of Hypotrichomonadea relative to Trichomonadea varies from one analysis to the other. Here, we present the first phylogeny of parabasalid Rpb1 sequences, in addition to updated analyses of our new parabasalid GAPDH, Pms1 and *EF1α* sequences compared to published data, and compare these results with revised phylogenetic analyses of published α-tubulin, malate dehydrogenase (MDH) and enolase sequence data.

### Rpb1 resolves the phylogeny of three groups of Parabasalia

We isolated *Rpb1* genes from 19 parabasalids and six other members of the Excavata by PCR using degenerate primers, and hemidegenerate reactions using one degenerate and one specific primer. All parabasalid primary PCR products were gel-isolated and sequenced directly without cloning, except for *Monotrichomonas carabina Rpb1*, where the template DNA quantity was low (<10 ng/µl) and derived from a non-axenic culture. Generally, the products from our simple PCR protocols were only cloned if the yield from PCR was insufficient for direct sequencing, usually attributed to a relatively scarce quantity of template DNA. Consistent with the single-copy status of the *Rpb1* gene in *Trichomonas vaginalis* isolates G3 and NIH:C1, the *Rpb1* genes we sequenced from other organisms also appear to be single-copy. Eight cloned PCR products of *Monotrichomonas carabina Rpb1* had identical sequences, consistent with a single-copy gene. No sequence ambiguities (double peaks in the electropherograms) were identified in any of the *Rpb1* PCR products directly sequenced from each parabasalid isolate with degenerate or specific sequencing primers.

**Figure 2:**
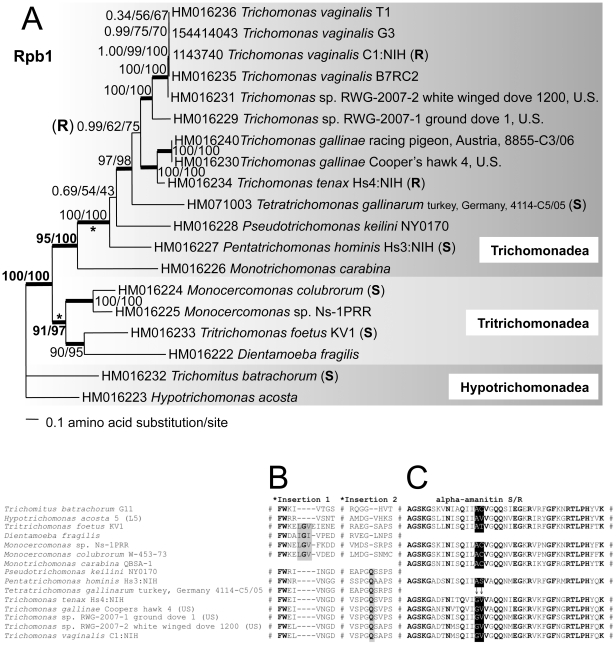
Rpb1 proteins resolve monophyletic Trichomonadea, Tritrichomonadea and Hypotrichomonadea, and species and isolates within these groups. All data are from this study, except *T. vaginalis* isolates G3 and NIH:C1. (**A**) The phylogenetic tree topology calculated by PhyML 3.0 from 1014 unambiguously aligned amino acids spanning conserved regions A to G of Rpb1 is shown (see **Figure S1**). Thickened lines indicate the nodes supported by a Bayesian posterior probability of 1.00. Numbers at the nodes correspond to Bayesian posterior probabilities, followed by percent bootstrap support ≥50% given by PhyML and RAxML (1000 replicates each), with LnL = −14857.5, α = 1.38, pI = 0.21. Scale bar represents 0.1 amino acid substitution per site. *Asterisks indicate relationships also supported by insertions. “**S**” indicates α-amanitin sensitivity, while “**R**” indicates resistance to α-amanitin [61]. (**B**) Conserved insertions in Rpb1 region A, with one unique insertion uniting Trichomonadea and another unique insertion only found in Tritrichomonadea. 100% identical aligned amino acids are shown in bold, gaps in the alignment indicated by dashes and #. (**C**) Conserved region E of Rpb1, which exhibits sensitivity to α-amanitin [67], [68]. Arrows indicate glycine and valine residues (A780G and C781V substitutions) that probably confer α-amanitin resistance to members of the *Trichomonas* genus. The complete Rpb1 alignment is provided in the **Dataset S1**. GenBank accession numbers are shown at the left for each taxon.

Our analyses of parabasalid Rpb1 proteins generated a fully resolved phylogeny of various isolates, species and genera (**Figure 2A**), within three classes that are consistent with prior rDNA studies [37], and concatenated α- and β-tubulin, enolase and GAPDH analyses of a few of the organisms [7], [11]. All *Trichomonas* specimens from three species form a monophyletic group, and are included in the Trichomonadea together with *Tetratrichomonas*, *Pseudotrichomonas*, *Pentatrichomonas* and *Monotrichomonas*. Interestingly, Rpb1 resolves the evolutionary relationships of some avian isolates consistent with the 18S rDNA and α-tubulin phylogenies of these isolates relative to *T. vaginalis*, *T. gallinae*, *T. tenax*, *Trichomonas* sp. and *Tetratrichomonas gallinarum* [59], and analyses of 5.8S rDNA and internally transcribed spacers [60]. In the Tritrichomonadea, *Dientamoeba fragilis* is most closely related to *Tritrichomonas foetus*, and this group is most closely related to *Monocercomonas colubrorum* and *Monocercomonas* sp. Ns-1PRR. *Trichomitus batrachorum* and *Hypotrichomonas acosta* are clearly united as members of the Hypotrichomonadea. Parabasalid Rpb1 sequences exhibit two conserved amino acid insertions (**Figure 2B**), which lend further support to the phylogenetic tree. One of these rare genomic events unites Trichomonadea, and the other is unique to Tritrichomonadea.

Comparative biochemistry of parabasalid Rpb1 proteins indicates that resistance to the transcription elongation inhibitor α-amanitin is limited to the genus *Trichomonas*, with variation in the degree of α-amanitin sensitivity of other Trichomonadea, Tritrichomonadea and Hypotrichomonadea [57], [61]. Conserved substitutions to glycine and valine at *T. vaginalis* Rpb1 amino acid positions 780 and 781 in the domain typically involved in polymerase translocation during transcription elongation in all eukaryotes (**Figure 2C**, details in **Figure S1**) suggest that parabasalid α-amanitin resistance evolved in the last common ancestor of the genus *Trichomonas*, and can be attributed to these two amino acid positions in the Rpb1 “α-amanitin binding pocket” described previously [62], [63], [64], [65], [66]. Typical eukaryotic α-amanitin sensitive Rpb1 proteins [65], [66], [67], [68] usually encode alanine and cysteine residues at those positions instead. We can infer from the Rpb1 phylogeny that *P. keilini*, *M. carabina*, *Monocercomonas* sp. Ns-1PRR, *D. fragilis*, and *H. acosta* would likely be sensitive to α-amanitin since their closest relatives are sensitive [61], and where the data are available these organisms lack the A780G and C781V substitutions found in *Trichomonas*.

Interestingly, our data indicates that *Rpb1* genes are intronless in the regions spanning conserved Rpb1 domains A through G in metamonads, *Parabodo caudatus*, *Diplonema* sp. 2 and *Percolomonas cosmopolitus*, while abundant predicted spliceosomal introns interrupt the open reading frames of *Naegleria gruberi*, *J. libera* and *Malawimonas Rpb1* genes. This characteristic makes a ∼3.1 kb PCR amplicon from the *Rpb1-*coding sequence a good target genetic marker for total DNA specimens from the intron-sparse subgroups of the Excavata, while future work may benefit from cDNA amplification of *Rpb1* from intron-rich organisms. However, additional data from intron containing excavate *Rpb1* genes is necessary for deducing patterns of intron loss or gain since the last common ancestor of all excavates.

**Figure 3:**
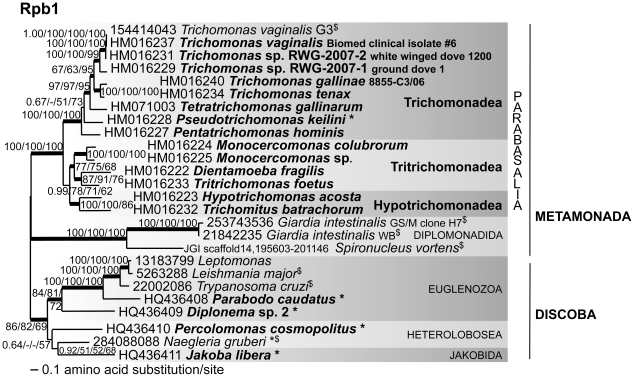
Rooted parabasalid Rpb1 phylogeny shows that Hypotrichomonadea are closer to Tritrichomonadea than to Trichomonadea. New sequences for this study are indicated in bold type, *indicates free-living species, and ^$^indicates data from a publicly available genome sequence. This tree topology was calculated by PhyML 3.0 from 936 unambiguously aligned amino acids spanning conserved regions A to G of Rpb1. Thickened lines indicate the nodes supported by a Bayesian posterior probability of 1.00. Numbers at the nodes correspond to Bayesian posterior probabilities from the best post burn-in 9500 trees, followed by percent bootstrap support ≥50% given by PhyML and RAxML (1000 replicates each), and parsimony (100 replicates, PAUP*). LnL = −26857.70, α = 1.43, pI = 0.088. Removal of constant sites did not change the topology or support in an additional RAxML analysis (results not shown). Metamonada is also recovered in majority-rule consensus topologies when a different outgroup is used (**Figure S2**). Scale bar represents 0.1 amino acid substitution per site. The complete Rpb1 alignment is provided in the **Dataset S1**. GenBank accession numbers or Joint Genome Institute locus ID are shown at the left for each taxon.

**Figure 4:**
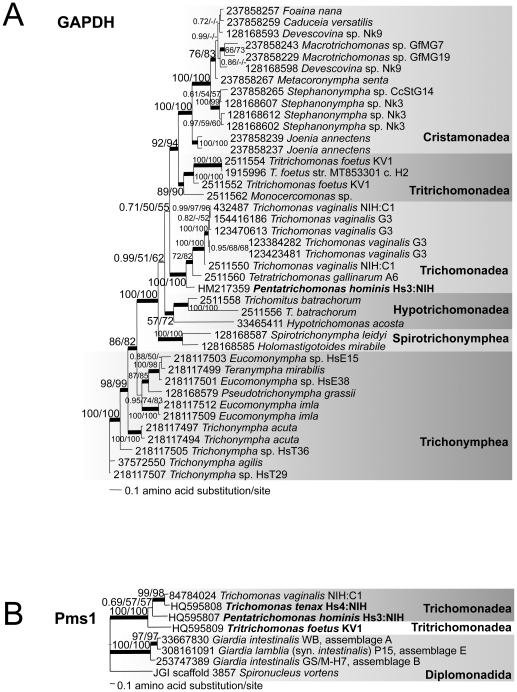
(A) GAPDH resolves six monophyletic parabasalid groups, but exhibits multiple nonidentical gene copies per taxon, while (B) Pms1 resolves Trichomonadea. The consensus tree topologies of the sets of best trees calculated by Bayesian inference are shown. Data generated in this study is highlighted by bold type. Scale bar represents 0.1 amino acid substitution per site. Thickened lines indicate the nodes supported by a Bayesian posterior probability of 1.00. Numbers at the nodes correspond to Bayesian posterior probabilities, followed by percent bootstrap support ≥50% given by PhyML and RAxML (1000 replicates each). The alignments are provided in **Dataset S3** (GAPDH) and **Dataset S4** (Pms1). (**A**) **GAPDH**. This consensus topology of the 8750 best trees calculated by Bayesian inference was constructed from 324 aligned amino acids. LnL = −7323.20, α = 1.06 (0.72<α<1.48), pI = 0.14 (0.053<pI<0.22). (**B**) **Pms1**. This consensus topology of the 9500 best trees was calculated by Bayesian inference from 538 aligned amino acids. LnL = −7126.44, α = 3.54 (2.70<α<3.98), pI = 0.040 (0.011<pI<0.071). GenBank accession numbers or Joint Genome Institute locus ID are shown at the left for each taxon.

**Figure 5:**
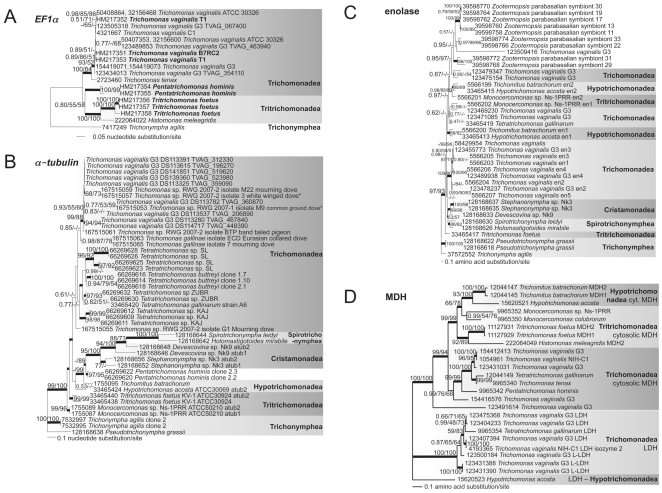
Phylogenetic analyses of parabasalid *EF1α*, *α-tubulin*, enolase and MDH exhibit discordant topologies and multiple nonidentical gene copies per taxon. The consensus tree topologies of the sets of best trees calculated by Bayesian inference are shown. Thickened lines indicate the nodes supported by a Bayesian posterior probability of 1.00. Numbers at the nodes correspond to Bayesian posterior probabilities, followed by percent bootstrap support ≥50% given by PhyML and RAxML (1000 replicates each). The alignments are provided in the **Datasets S5** (*EF1α*), **S6** (*α-tubulin*), **S7** (enolase) and **S8** (MDH). (**A**) ***EF1α***. *T. foetus*, *P. hominis* and *T. vaginalis EF1α* sequences determined in this study are indicated in bold. 1230 nucleotides partitioned by codons were analyzed, giving this consensus topology of the 9250 best trees. LnL = −5053.71, α = 2.66 (1.21<α<5.26), pI = 0.043 (0.0015<pI<0.13). Scale bars represent 0.05 nucleotide substitution per site. (**B**) ***α-tubulin***. This consensus topology of the 8000 best trees was drawn from 1041 aligned nucleotides that were partitioned by codons. LnL = −10690.49, α = 1.27 (1.02<α<1.59), pI = 0.018 (0.00083<pI<0.049). Scale bar represents 0.1 nucleotide substitution per site. *indicate the same *Trichomonas* sp. from which we also obtained *Rpb1* genes. (**C**) **Enolase**. Analysis of 331 aligned amino acids gave this consensus topology of the 8250 best trees. LnL = −9781.32, α = 0.84 (0.70<α<1.00), pI = 0.013 (0.00034<pI<0.044). Scale bar represents 0.1 amino acid substitution per site. (**D**) **MDH**. 308 amino acids were analyzed, giving this consensus topology of the 9000 best trees. LnL = −6019.55, α = 1.48 (1.07<α<2.07), pI = 0.049 (0.0032<pI<0.11). Scale bar represents 0.1 amino acid substitution per site. GenBank accession numbers are shown at the left for each taxon.

Our phylogenetic tree of Rpb1 from parabasalids and other excavates rooted with *Jakoba libera* (Discoba) as the outgroup is shown in **Figure 3** (inferred from data in **Dataset S1**). Similar to recent phylogenies of multiple concatenated proteins [5], this analysis of Rpb1 also resolves Metamonada (Parabasalia, Preaxostyla (not shown) and Fornicata, represented here by the diplomonads *Giardia* and *Spironucleus*) distinct from the Discoba. Analyses of Rpb1 with and without constant sites, and with different outgroups also recover Metamonada in the majority-rule consensus topology, indicating that Discoba are at least as good as any other outgroup to Metamonada (**Figure S2** and **Dataset S2**, and results not shown). The rooted analysis of Rpb1 in **Figure 3** indicates that Hypotrichomonadea is more closely related to Tritrichomonadea than it is to Trichomonadea, a specific relationship that remains to be borne out once additional Rpb1 data is acquired from fresh isolates of uncultivable parabasalids from Cristamonadea, Spirotrichonymphea and Trichonymphea. While the relationship of Tritrichomonadea to Hypotrichomonadea shown in **Figure 3** is inconsistent with results of our analyses of GAPDH and other proteins (**Figures 4** and **5**), it is consistent with relationships seen with some enolase and MDH paralogs (**Figure 5**).

### Morphology

The relationship between Tritrichomonadea and Hypotrichomonadea that we observe in the Rpb1 phylogeny (**Figure 3**) is also consistent with one morphological (synapomorphic) character shared only by some members of both of these groups. The undulating membranes of *Tritrichomonas foetus* (Tritrichomonadea) and *Trichomitus batrachorum* (Hypotrichomonadea) are both supported by a costa comprised of A-type fibers [69], [70], while other members of these groups have a reduced costa (*Hypotrichomonas* [71]) or lack a costa altogether (*Monocercomonas* [72], *Dientamoeba* [73]), and the costae of Trichomonadea (*e.g.*, *Pentatrichomonas*, [74]) are structurally arranged as B-type fibers though also considered homologous [36]. If the hypothesis that some devescovines (within Cristamonadea) also have a remnant A-type costa is correct (discussed by [11] and references therein), then it is possible that A-type costae were ancestral to the group comprised of Tritrichomonadea, Cristamonadea and Hypotrichomonadea. Further ultrastructural and molecular phylogenetic analysis of putative basal lineages in this group such as *Trichocovina*, which has a costa [75], might support this hypothesis. Simpson and Patterson noticed a striking similarity between the arrangement of B-type fibers in the parabasalian costa and the C-fibers of the jakobid flagellar apparatus, and proposed the hypothesis that these structures are homologous, a synapomorphy uniting the Parabasalia with Excavata [1], [76]. This hypothesis is now supported by phylogenomic studies that support the position of Parabasalia in Excavata [2], [5], [6]. Further conclusions are precluded pending scrutiny of ultrastructural characters of a more diverse sample of Tritrichomonadea and Hypotrichomonadea in comparison with basal free-living lineages of the Trichomonadea (*i.e.*, *Monotrichomonas*).

### Trichomonadea, Hypotrichomonadea, and GAPDH

We sequenced *GAPDH* from *Pentatrichomonas hominis* (Trichomonadea) and analyzed all available parabasalid GAPDH predicted protein sequences in GenBank, with and without an outgroup (**Dataset S3**). Our GAPDH analyses assign the same genera to the six groups of Parabasalia as previously published GAPDH analyses [7], [8], [9], [49], with modest to high support for the monophyly of each group, and usually for the relationships of genera within the groups (**Figure 4A**). We also analyzed parabasalid GAPDH homologs rooted with their closest relatives in Preaxostyla and Bacteria, with constant sites removed (**Figure S3**), hoping to identify the position of the root of the parabasalid tree. Relationships between the six parabasalid groups were unsupported except by Bayesian analysis in the rooted tree, except for the resolution of Cristamonadea as most closely related to Tritrichomonadea. Hypotrichomonadea often appear to be related as a sister to the Trichomonadea in molecular phylogenies of 18S rDNA, GAPDH, enolase, α-tubulin and analyses of concatenated sequences [7], [8], [9], [11], [38], a relationship also recovered in the majority-rule consensus topology of our rooted analysis. The addition of GAPDH from *Pentatrichomonas hominis* (**Figure 4A**) has changed the unrooted tree topology, and we no longer see this specific sister relationship between the Trichomonadea and Hypotrichomonadea. Instead, the unrooted GAPDH phylogeny resolves Trichomonadea as the closest relative to a clade comprised of the Cristamonadea and Tritrichomonadea with some support (**Figure 4A**). This relationship merits further attention by expanding the taxon sampling of GAPDH in Trichomonadea beyond three genera to include basal free-living lineages such as *Pseudotrichomonas* and *Monotrichomonas*. Our unrooted phylogeny of GAPDH also differs markedly from the most recent concatenated analysis of 18S rDNA and GAPDH, enolase, and α- and β-tubulin proteins in the close relationship of the Spirotrichonymphea and Cristamonadea (with less than 50% support) [11]. The results of the unrooted phylogeny (**Figure 4A**) indicate that Spirotrichonymphea is a sister to the Trichonymphea with moderate support, consistent with prior analyses of GAPDH.

### Pms1 is a useful genetic marker in preliminary analyses

**Figure 4B** illustrates our phylogenetic analysis of another protein coded by a single-copy gene, Pms1, which is a mismatch repair protein homologous to prokaryotic mutL and is conserved in all eukaryotes [56]. We sequenced *Pms1* genes from *Trichomonas tenax*, *Pentatrichomonas hominis* and *Tritrichomonas foetus*. We recently demonstrated that *Pms1* genes in *T. vaginalis* isolates commonly cultured in the laboratory are genetically diverse and useful for phylogenetic analysis of conspecific isolates [54]. Our phylogenetic analysis of more diverse parabasalid Pms1 proteins rooted with diplomonads as the outgroup indicates that Pms1 will also be a useful genetic marker for resolving parabasalid relationships at the genus and species level, and provides modest support for distinguishing Trichomonadea as a group apart from *Tritrichomonas foetus* in this pilot study. Unlike *Rpb1* genes, we did not amplify *Pms1* genes from parabasalids using universal eukaryotic degenerate PCR primers. However, we did isolate the *T. tenax Pms1* gene using degenerate oligonucleotides designed from the specific amino acid sequences of *T. vaginalis* Pms1 in conserved regions of a eukaryotic Pms1 multiple sequence alignment. Inspection of Pms1 amino acid sequences of Trichomonadea and *T. foetus* aligned with Pms1 from diplomonads *Giardia intestinalis* (syn. *lamblia*) and *Spironucleus vortens* (**Dataset S4**) indicates that future genetic studies might exploit conserved parabasalid Pms1 amino acid motifs DNG(P/C)GI and PWNCPGH for the design of specific parabasalid degenerate forward and reverse Pms1 PCR primers for an approximately 1.5 kb amplicon, but that experiment is beyond the scope of this study.

### EF1α preliminary analyses reveal paralogy

We identified homologs of *EF1α* genes from the databases and by degenerate PCR, to evaluate the usefulness of this ubiquitous protein-coding gene for resolving the relationships of *T. vaginalis* isolates and different species and genera of parabasalids (**Figure 5A** and **Dataset S5**). We isolated and sequenced eight *EF1α* genes by PCR and assembled three others from expressed sequence tags (from dbEST). Tritrichomonadea was resolved as a monophyletic group but Trichomonadea was not. Relationships of *EF1α* paralogs and orthologs from five *T. vaginalis* isolates (G3, C1:NIH, T1, B7RC2 and ATCC30326) were poorly resolved with this gene. *EF1α* genes appear to be recently duplicated within the lineages of *Tritrichomonas foetus*, *Pentatrichomonas hominis* and *T. vaginalis*. Since our degenerate PCR amplicons always yielded several distinct sequences including these paralogs, and the phylogeny did not resolve *P. hominis* among the Trichomonadea, we did not develop *EF1α* further as a phylogenetic marker for Parabasalia.

### Other protein-coding genes

We re-analyzed published sequences of available homologs of other protein-coding genes conventionally used to infer parabasalid phylogenies available as of June 2010, to evaluate their usefulness for phylogenetic resolution especially at the species and isolate level. These revised analyses of published *α-tubulin* genes and MDH and enolase amino acid sequences offer somewhat better resolution than prior analyses of these genes with fewer organisms, but they do not resolve the six monophyletic parabasalid groups (**Figure 5** and **Datasets S6, S7** and **S8**). We examined the relationships of multiple copies of these genes encoded in the genome sequence of *Trichomonas vaginalis* G3 (all on different scaffolds) with available data from other *T. vaginalis* isolates that lack a complete genome sequence. Recent analyses of β-tubulin continue to reveal pervasive duplication in diverse parabasalids and fail to resolve the six parabasalid groups [49], [77], consistent with our β-tubulin analysis (not shown). Thus α- and β-tubulin, MDH and enolase sequences do not appear to be useful for resolving the relationships of *T. vaginalis* isolates since they appear prone to phylogenetic artifacts arising from comparisons of non-orthologous paralogs unless all the paralogs from each isolate are identified and sequenced. The genes encoding these proteins usually are not variable enough to permit the design of paralog-specific PCR primers. Since most investigations into the relationships among isolates rely on high-throughput sensitive and accurate PCR-based approaches to isolating and sequencing orthologous loci, these genes appear impractical for further pursuit in that direction. Enolase is known to exhibit phylogenetic discordance because of recombination [45]. While prior analyses with fewer genera indicated that α- and β-tubulin paralogy does not interfere with their ability to resolve the relationships among parabasalid genera in concatenated analyses [7], our analysis indicates that increased taxon sampling does not improve the resolution among genera or classes at a level comparable to Rpb1, GAPDH or rDNA phylogenies.

### Conclusions

Genetic analysis of eukaryotic microorganisms is an increasingly common technique for establishing their relationships, with major impacts on their taxonomy. The use of a small unique part of the genome, such as a single-copy gene, as a genetic marker offers a straightforward approach for elucidating the phylogenetic position of diverse parabasalids. Rpb1 amino acid sequences proved useful in resolving parabasalid relationships at various levels of taxonomic resolution, *i.e.*, isolate, species, genus and upward. Improved taxon sampling of *Rpb1* genes from metamonads and other protists will help resolve the placement of Parabasalia in the evolutionary tree of eukaryotes with even greater confidence. GAPDH is also useful for resolving relationships beyond the genus level within Parabasalia, and could be a useful marker for determining the position of the root of the Parabasalid tree with expanded taxon sampling and using closely-related Preaxostyla as the outgroup. *Pms1* genes are also potentially useful for resolving higher taxonomic levels within the Parabasalia. Owing to pervasive duplication or recombination, genes coding for tubulin, MDH, EF1α and enolase proteins [45] should be abandoned as phylogenetic markers within the Parabasalia, and efforts shifted towards Rpb1, which is also useful to compare Parabasalia with all other eukaryotes. *Rpb1* and *Pms1* genes behave like a single-copy gene in all of the parabasalids included in the study, regardless of their genome size. Furthermore, *Rpb1* exhibits specific conserved insertions diagnostic of Trichomonadea and Tritrichomonadea. Our recent analysis of microsatellites and other single-copy genes demonstrated genetic diversity among *T. vaginalis* isolates [54], consistent with results presented here. Thus far, *Rpb1* is the only protein-coding gene that has been isolated and sequenced directly using degenerate primers (without requiring cloning) from diverse cultivable Parabasalia. Rpb1 exhibits enough informative substitutions between isolates, species, and beyond that it should be useful for inferring the evolutionary relationships of other genetically diverse parabasalids, and their close relatives.

## Materials and Methods

### Database searches

Keyword searches of the National Center for Biotechnology Information (NCBI) protein and nucleotide non-redundant database revealed homologs of Rpb1, GAPDH, enolase, MDH, α- and β-tubulin, Pms1 and EF1α. Their DNA and inferred protein sequences were used as queries for BLASTn and BLASTp searches [78] of parabasalid homologs in the database of non-human non-mouse expressed sequence tags (dbEST-other) and the NCBI nonredundant database. These BLASTP searches were extended to the publicly available databases of the Joint Genome Institute (*Spironucleus* Rpb1 and Pms1, and *Emiliania* and stramenopile Rpb1), the Broad Institute (*Capsaspora* and *Thecamonas* Rpb1) and NCBI to retrieve representative outgroup sequences for Rpb1, GAPDH and Pms1 proteins.

We also obtained partial gene sequence data for *Pentatrichomonas hominis* and *Tritrichomonas foetus Rpb1*, *EF1α*, *Pms1* and *P. hominis GAPDH* genes from preliminary 2.5× coverage genomic shotgun sequencing (Roche 454 Technologies) at NYU Langone Medical Center's Genome Technology Core. We used DNA and inferred protein sequences from GenBank or our own degenerate PCR results as queries for local BLASTn and tBLASTn searches of the nucleotide sequence assemblies to identify sequences.

### Sources of DNA templates

Study organisms are summarized in **Table 1**. Cells of *Tritrichomonas foetus* KV-1 (American Type Culture Collection (ATCC) #30924, Mannassas VA, USA) were cultured axenically at 37°C in Diamond's TYM medium [79] pH 7.2 supplemented with 10% fetal bovine serum and 0.1 U/ml penicillin-streptomycin (Invitrogen, Carlsbad CA, USA). *T. vaginalis* isolates CI6 [54] and B7RC2 were cultured similarly in TYM medium [79] at pH 6.2 and supplemented with 10% horse serum (Invitrogen, Carlsbad CA, USA) instead. Total DNA was extracted by disrupting the cells in UNSET buffer followed by phenol-chloroform extraction and isopropanol precipitation [80]. Genomic DNA was prepared using the same method from *P. hominis* cultured axenically by Shelby Bidwell at 37°C in Diamond's LYI medium [15] supplemented with 10% bovine serum and 0.1 U/ml penicillin-streptomycin (Invitrogen, Carlsbad CA, USA). Total DNA was similarly prepared directly from frozen stabilates of *H. acosta*, *T. batrachorum*, *M. colubrorum* and *Monocercomonas* sp. Clonal cultures of *T. gallinae*/Racing pigeon/Austria/8855-C3/06 and *T. gallinarum*/Turkey/Germany/4114-C5/05 were established, axenized, propagated and DNA prepared as described [60], [81], [82].

**Table 1:** Study organisms used in this project.

Species	Isolate	Availability	Isolated from	Xenic/axenic	Ref.
*Trichomonas vaginalis*	B7RC2	ATCC #50167	human vagina, Greenville NC USA	axenic	[102]
*Trichomonas vaginalis*	T1	Jane Carlton	human vagina, Taipei, Taiwan	axenic	[103]
*Trichomonas vaginalis*	CI6	BioMed Diagnostics	human vagina, Puerto Rico	axenic	[54]
*Trichomonas* sp. RWG-2007-2	WWDO1200	Rick Gerhold	white winged dove, USA	axenic	[59]
*Trichomonas* sp. RWG-2007-1	CGDO1	Rick Gerhold	common ground dove, USA	axenic	[59]
*Trichomonas tenax*	Hs-4:NIH	ATCC #30207	human mouth	axenic	[15]
*Trichomonas gallinae*	COHA4	Rick Gerhold	Cooper's Hawk, USA	axenic	[59]
*Trichomonas gallinae*	8855-C3/06	Michael Hess, VMU	racing pigeon, Austria	axenic	[60]
*Tetratrichomonas gallinarum*	4114-C5/05	Michael Hess, VMU	turkey, Germany	axenic	[82]
*Pentatrichomonas hominis*	Hs-3:NIH	ATCC #30000	human intestine, Korea	axenic	[104]
*Pseudotrichomonas keilini*	NY0170	ATCC #PRA-328	free-living, mangrove sediments, Japan.	xenic	[37]
*Monotrichomonas carabina*	QBSA-1	ATCC #50700	free-living	xenic	[41]
*Monocercomonas colubrorum*	W-453-73	ATCC #30225	lizard (*Tupinambis teguixin*), ON Canada	axenic	[105]
*Monocercomonas* sp.	Ns-1PRR	ATCC #50210	snake (*Natrix sipedon*), MD USA	axenic	[105]
*Tritrichomonas foetus*	KV-1	ATCC #30924	*Bos taurus*, Czech Republic	axenic	[106]
*Dientamoeba fragilis*	G	John Ellis, U. of Sydney	human stool, Australia (culture died)	xenic	[83]
*Trichomitus batrachorum*	G 11	ATCC #30066	snake (*Elaphe obsolete*), Bronx Zoo, NY USA	axenic	[105]
*Hypotrichomonas acosta*	5 (L5)	ATCC #30070	snake *Crotalus* sp., Argentina	axenic	[105]
*Diplonema* sp. 2	IIIGPC	ATCC #50224	free-living, marine aquarium MD USA	axenic	[107]
*Parabodo caudatus*	RCP	ATCC #50361	free-living, sediment near shore, MD USA	xenic	[108]
*Percolomonas cosmopolitus*	AE-1	ATCC #50343	free-living, marine aquarium MD USA	xenic	[109]
*Jakoba libera*	CB	ATCC #50422	free-living, deep marine sediments	xenic	[110]
*Malawimonas jakobiformis*	AF2	ATCC #50310	free-living, Lake Malawi enriched sediment	xenic	[111]
*‘Malawimonas californiana’*	CA-1	ATCC #50740	free-living, California enriched sediment	NK	[5]

Dr. Patricia Johnson (University of California – Los Angeles) provided genomic DNA for *T. tenax*, and *T. vaginalis* isolate T1. Jeff Cole and Robert Molestina (ATCC) provided the genomic DNA of *M. carabina*, *P. caudatus*, *Diplonema* sp. 2, *J. libera*, *P. cosmopolitus* and *Malawimonas*. Dr. Naoji Yubuki (University of British Columbia) provided genomic DNA of freshly isolated *P. keilini* [37]. Dr. John Ellis (University of Technology – Sydney, Australia) provided genomic DNA from *D. fragilis* [83]. Rick Gerhold and Dr. Larry McDougald (University of Georgia) provided genomic DNA of U.S. *T. gallinae* isolate COHA4 and *Trichomonas* sp. isolates WWDO1200 and CGDO1 [59].

### PCR conditions and amplicon sequencing

Rpb1 amino acid sequences were obtained from GenBank, aligned using MUSCLE v. 3.7 [84], [85] and alignments adjusted manually using MacClade 4.08 [86] (**Datasets S1** and **S2**). We designed degenerate forward and reverse oligonucleotides **(Tables S1** and **S2)** based upon conserved amino acid sequence motifs in the multiple sequence alignment (**Figure S1**), with reference to published Rpb1 PCR primers [58]. Relative to *T. vaginalis*, degenerate primers Rpb1AF1 *vs* Rpb1GR1 correspond to a ∼3.1 kb PCR product in Parabasalia. We designed PCR primers specific to *T. vaginalis Rpb1* from isolates NIH:C1 and G3 (NCBI GI# 1143739 and 154414042, and **Table S1**). Once we collected *Rpb1* sequences from a few parabasalid genera, amino acid sequences were aligned and additional internal degenerate and specific primers designed to use for sequencing reactions and PCR (**Table S1**). Degenerate and specific primers listed in **Table S2** were used to amplify and sequence *P. caudatus*, *Diplonema* sp. 2, *J. libera*, *P. cosmopolitus* and *Malawimonas Rpb1* genes. Amplicons obtained by primary degenerate PCR were often extended by hemidegenerate PCR to obtain longer *Rpb1* sequences.

Combinations of degenerate and *T. vaginalis*-specific primers were used to amplify parabasalid *Rpb1* homologs by PCR. The most useful primer combinations for primary PCR amplification of diverse new parabasalid *Rpb1* genes were degenerate primers Rpb1AF1 *vs* Rpb1GR1 (∼3.1 kb amplicon), and degenerate Rpb1AF1 *vs T. vaginalis*-specific TvRpb1DR (∼1.2 kb amplicon). If the Rpb1AF1 *vs* TvRpb1DR combination proved more useful, then after sequencing the PCR product we paired Rpb1GR1 *vs.* a specific forward primer designed from the 3′ end of the PCR product to amplify the remaining ∼1.8 kb by hemidegenerate PCR.

*Trichomonas vaginalis Pms1* degenerate oligonucleotides TvPms1dF2 (forward, 5′ ATGAAGACGCTGRGYAARCAYGA 3′) *vs.* TvPms1dR1 (reverse, 5′ GTCGGTCTACCGTGCGGRCARTTCCANGG 3′) were used to generate and then sequence an ∼1.6 kb PCR amplicon of the *Trichomonas tenax Pms1* gene. Reverse primers TtxPms1SR1 (5′ GACTGGTTCCATTGTCC 3′) and TtxPms1SR2 (5′ GAATTAGTCGTTGGTGACGC 3′) were used for internal sequencing. *EF1α* genes were amplified and sequenced with described degenerate primers 1XF *vs.* 10XR [87].

We amplified genes from total DNA by PCR with 5Prime MasterTaq™ DNA polymerase (Hamburg, Germany) and Stratagene Cloned Pfu™ DNA polymerase (La Jolla CA, USA), as recommended by the manufacturers, with ∼10–40 ng DNA, 250 µM each dNTP (Fermentas, Glen Burnie MD USA), 1.5 mM MgCl_2_ and 1 µM each primer (synthesized by Eurofins MWG Operon [Hunstville AL, and Ebersberg, Germany], or by Integrated DNA Technologies (IDT), Coralville IA, USA) per reaction. We amplified *Rpb1* genes of *T. gallinae* 8855-C3/06 and *T. gallinarum* 4114-C5/05 isolates from 20 ng of total DNA with the Qiagen HotStarTaq™ Master Mix Kit (Vienna, Austria), as directed by the manufacturer. Reaction conditions were 95°C for 3 minutes followed by 40 cycles at 94°C for 30 seconds, 45, 50 or 55°C for 1 minute and 72°C for 2 or 3 minutes+6 seconds/cycle, then ending at 72°C for 10 minutes. *M. carabina*, *Diplonema* sp. 2, *J. libera* and *P. cosmopolitus Rpb1* thermocycling conditions were 95°C for 3 minutes followed by 40 cycles at 92°C for 90 seconds, 45, 50, 55 or 60°C for 90 seconds and 72°C for 3 or 5 minutes+6 seconds/cycle, then ending at 72°C for 10 minutes. We fractionated PCR products by agarose gel electrophoresis (0.8% agarose with 1× TAE buffer run for 60 minutes at 110 V), visualized by ethidium bromide staining, excised, and then purified them with the Promega Wizard™ Gel Isolation Kit (Madison WI, USA) and QIAquick™ Gel Extraction Kit (Qiagen, Vienna, Austria).

Internal sequencing primers were typically necessary since most amplicons were too large to be adequately covered by only sequencing their ends. We sequenced most PCR products directly by primer walking using BigDye™ 3.1 technology (Applied Biosystems (ABI), Foster City CA, USA). Sequencing reactions were purified using CleanSeq™ magnetic beads (Beckman-Coulter, Beverly MA, USA), and run on an ABI 3130*xl*™ or ABI 3730™ instrument (ABI, Foster City CA, USA). *T. gallinae* isolate 8855 clone C3/06 and *T. gallinarum* isolate 4114 clone C5/05 PCR products were sequenced similarly by Eurofins MWG Operon (Ebersberg, Germany).

A few *Rpb1* PCR amplicons obtained from DNAs of low concentration (<10 ng/µl) that were prepared from non-axenic cultures were cloned, since the PCR amplicon yield was too low to be sequenced directly. These included *Rpb1* genes of a single parabasalid (*M. carabina*) and six other excavates.

Prior to sequencing, we cloned *Rpb1* PCR amplicons from *M. carabina* (conserved regions D through G, ∼1.8 kb), and various overlapping degenerate and hemidegenerate *Rpb1* PCR amplicons from *P. caudatus*, *Diplonema* sp. 2, *J. libera*, *P. cosmopolitus* and *Malawimonas*. We fractionated PCR amplicons electrophoretically in 0.5–0.75% low melt: 0.5–0.75% NuSieve™ GTG agarose (Fisher, Pittsburgh PA, and BioWhittaker, Walkersville MD, USA), excised bands and cloned them directly into the pSC-A™ vector (StrataClone™ kit, Stratagene, La Jolla CA, USA) according to the manufacturer's instructions. We screened transformants by the size of their plasmid inserts by PCR with M13 forward *vs* reverse primers, cycling at 94°C for 2 minutes followed by 30 cycles at 94°C for 1 minute, 57°C for 2 minutes and 72°C for 90 seconds, then ending at 72°C for 5 minutes [88]. PCR reagents were as indicated above, including Taq DNA polymerase from New England Biolabs (Ipswich MA, USA) and Fisher (Pittsburgh PA, USA). We isolated (Eppendorf FastPlasmid™ Kit, Hamburg Germany) and sequenced selected clones as described above using M13 forward and reverse and gene-specific primers (IDT, Coralville IA, USA).

We assembled sequences and annotated putative open reading frames by using Sequencher™ 4.8 (Genecodes, Ann Arbor MI, USA) with reference to pairwise comparisons made by BLASTx of GenBank and to multiple sequence alignments of homologous proteins made with MUSCLE v. 3.7 [84], [85]. Where applicable, vector and PCR primer sequences were excluded from the assemblies. All sequences determined in this study have been deposited in GenBank and assigned accession numbers HM016222–HM016241, HQ436408–HQ436411, HQ834947 and HQ834948 for *Rpb1*, HM071003 for *GAPDH*, HQ595807–HQ595809 for *Pms1*, and HM217351–HM217359 for *EF1α*.

### Phylogenetic analysis

We used phylogenetic analyses to infer the evolutionary relationships of *Rpb1* and other protein-coding genes. We initially constructed multiple alignments of amino acid sequences using MUSCLE v. 3.7 [84], [85], then inspected and adjusted them manually using MacClade 4.08 [86]. We only used unambiguously aligned amino acid sites or codons for phylogenetic analyses. Alignments including our new data are provided in **Datasets S1, S2, S3, S4, S5, S6, S7** and **S8**.

We used MrBayes v. 3.12 [89], [90], PhyML [91], [92], and RAxML v. 7.0.4 or 7.2.7 [93], [94] for phylogenetic analyses, hosted by the University of Oslo Bioportal ([95], http://www.bioportal.uio.no/), the CIPRES Science Gateway Portal v. 1.0, v. 2.2, and v. 3.1 at the San Diego Supercomputer Center ([96], http://www.phylo.org/portal/), or the South of France Bioinformatics Platform (http://www.atgc-montpellier.fr/phyml/). We ran MrBayes for 10^7^ generations, with four incrementally heated Markov chains, a sampling frequency of 10^3^ generations and the temperature set at 0.5. Among-site substitution rate heterogeneity was corrected using an invariable and eight gamma-distributed substitution rate categories and either the general time reversible (GTR) model of nucleotide substitutions or the WAG model for amino acid substitutions [97], abbreviated herein as GTR+I+8γ or WAG+I+8γ. The consensus tree topology, the arithmetic mean log-likelihood (lnL) for this topology, and branch support were estimated from the set of sampled trees with the best posterior probabilities. Means and 95% confidence intervals for the gamma distribution shape parameter (α) and the proportion of invariable sites (pI) were also estimated for each alignment that was analyzed. We analyzed Rpb1 proteins with PhyML v. 3.0 for 1000 bootstrap replicates using the LG model for amino acid substitutions [98], [99] (LG+I+8γ); other proteins were analyzed similarly or using WAG+I+8γ in PhyML v. 2, for **Figures 4** and **5**. Amino acid sequence phylogenies computed using RAxML v. 7.0.4 or RAxML v. 7.2.7 utilized the WAG+I+8γ or LG+I+8γsubstitution models for 1000 bootstrap replicates at the CIPRES Science Gateway Portal v. 1.0 or v. 3.1 at the San Diego Supercomputer Center ([100], http://www.phylo.org/portal/). Protein-coding nucleotide sequence alignments of *EF1α* and α-tubulin were analyzed using the GTR+I+8γ substitution model in all three programs. Finally, the Rpb1 amino acid alignment comprised of parabasalids, diplomonads and Discoba was also subject to 100 bootstrap replicates of maximum parsimony analysis using PAUP* v. 4.0b10 with the default settings [101].

## Supporting Information

Figure S1 **Alignment of Parabasalid Rpb1 proteins indicating conserved regions A–H.** Conserved regions A–H are underlined. 100% identical amino acid residues indicated in **bold**, conserved insertions highlighted in grey, and the a-amanitin sensitive region highlighted by a black box. Dashes indicate gaps or missing data. Arrows indicate the positions of PCR primers. Amino acid positions are indicated numerically in parentheses.
(PDF)

Figure S2 **Rooted eukaryotic Rpb1 phylogeny with constant sites removed recovers monophyletic Metamonada topology.** New sequences from this study are indicated in bold type. This tree topology was calculated by RAxML 7.2.7 from 857 unambiguously aligned amino acids spanning conserved regions A to G of Rpb1, with constant sites removed. Thickened lines indicate the nodes supported by a Bayesian posterior probability of 1.00. Numbers at the nodes correspond to Bayesian posterior probabilities from the best post burn-in 1500 trees (chains run for 2×10^6^ generations), followed by percent bootstrap support ≥50% given by PhyML (100 replicates) and RAxML (1000 replicates). LnL = −56091.16, α = 1.33, pI = 0.0013. Scale bar represents 0.1 amino acid substitution per site. The alignment is provided in the **Dataset S2**. GenBank accession numbers, Joint Genome Institute or Broad Institute locus IDs are shown at the left for each taxon.
(PDF)

Figure S3 **GAPDH phylogeny rooted with Preaxostyla and Bacteria does not resolve interrelationships of six parabasalid groups.** This consensus topology of the 9500 best trees calculated by Bayesian inference was constructed from 254 unambiguously aligned amino acids, with constant sites removed. LnL = −8779.10, α = 1.52 (1.25<α<1.83), pI = 0.0039 (0.000094<pI<0.014). Scale bar represents 0.1 amino acid substitution per site. Thickened lines indicate the nodes supported by a Bayesian posterior probability of 1.00. Numbers at the nodes correspond to Bayesian posterior probabilities, followed by percent bootstrap support ≥50% given by PhyML and RAxML (1000 replicates each). Data generated in this study is highlighted by bold type. The alignment is provided in the **Dataset S3**. Genbank accession numbers are shown at the left for each taxon.
(PDF)

Table S1 **Primers used to amplify and sequence fragments of parabasalid** ***Rpb1*** **genes.** Primers are listed from 5′ to 3′ positions within the gene.
(PDF)

Table S2 **Primers used to amplify and sequence fragments of Discoba** ***Rpb1*** **genes.** Primers for *Parabodo caudatus*, *Diplonema* sp. 2, *Percolomonas cosmopolitus*, *Jakoba libera* and *Malawimonas* are listed from 5′ to 3′ positions within the gene.
(PDF)

Dataset S1 **Complete alignment of excavate Rpb1 protein sequences in FASTA format.**
(FASTA)

Dataset S2 **Alignment of Rpb1 data used for Figure S2, in NEXUS format with MrBayes command block.** Ambiguously aligned or constant sites are removed.
(NEXUS)

Dataset S3 **Complete alignment of GAPDH protein sequences from Parabasalia, Preaxostyla and Bacteria, in NEXUS format.**
(NEXUS)

Dataset S4 **Complete alignment of metamonad Pms1 protein sequences in FASTA format.**
(FASTA)

Dataset S5 **Complete alignment of parabasalid** ***EF1α*** **nucleotide sequences in FASTA format.**
(FASTA)

Dataset S6 **Complete alignment of parabasalid** ***α-tubulin*** **nucleotide sequences in NEXUS format.**
(NEXUS)

Dataset S7 **Complete alignment of parabasalid enolase protein sequences in FASTA format.**
(FASTA)

Dataset S8 **Complete alignment of parabasalid MDH protein sequences in FASTA format.**
(FASTA)
