# Supplementary material for: Phylogeny of Parasitic Parabasalia and Free-Living Relatives Inferred from Conventional Markers vs. Rpb1, a Single-Copy Gene
Source: PLoS One. 2011 Jun 9;6(6):e20774. doi: 10.1371/journal.pone.0020774 (PMC3111441; doi:10.1371/journal.pone.0020774)
Supplement: Figure S1 — Alignment of Parabasalid Rpb1 proteins indicating conserved regions A–H. Conserved regions A–H are underlined. 100% identical amino acid residues indicated in bold, conserved insertions highlighted in grey, and the a-amanitin sensitive region highlighted by a black box. Dashes indicate gaps or missing data. Arrows indicate the positions of PCR primers. Amino acid positions are indicated numerically in parentheses. (PDF) [file pone.0020774.s001.pdf]

Supporting Information for:

Phylogeny of parasitic Parabasalia and free-living relatives inferred from conventional markers *vs. Rpb1,* a single-copy gene

(*PLOS One,* 2011. doi:10.1371/journal.pone.0020774)

Shehre-Banoo Malik[1,2*§], Cynthia D. Brochu[2], Ivana Bilic[3], Jing Yuan[2], Michael Hess[3], John M. Logsdon Jr.[2], and Jane M. Carlton[1§]

[1] Department of Microbiology – Division of Medical Parasitology, New York University Langone Medical Center, New York NY, United States of America.

[2] Department of Biology – Roy J. Carver Center for Comparative Genomics, University of Iowa, Iowa City IA, United States of America.

[3] Department for Farm Animals and Veterinary Public Health – Clinic for Avian, Reptile and Fish Medicine, University of Veterinary Medicine, Vienna, Austria.

[*] current address:
Department of Biochemistry and Molecular Biology – Center for Comparative Genomics and Evolutionary Bioinformatics, Dalhousie University, Halifax NS, Canada.

[§] Corresponding Authors

E-mail addresses:
SBM: sbmalik@dal.ca
CDB: cindy-brochu@uiowa.edu
IB: Ivana.Bilic@vetmeduni.ac.at
JY: yuanjing2003@hotmail.com
MH: Michael.Hess@vetmeduni.ac.at
JML: john-logsdon@uiowa.edu
JMC: jane.carlton@nyumc.org

Figure S1: Alignment of Parabasalid Rpb1 proteins indicating conserved regions A-H (underlined), with 100% identical amino acid residues indicated in **bold**, conserved insertions highlighted in grey, and the α-amanitin sensitive region highlighted by a black box. Dashes indicate gaps or missing data. Arrows indicate the positions of PCR primers. Amino acid positions are indicated numerically in parentheses.

```
                                                                                                                                     RPB1AF1>
Trichomitus batrachorum                                      --------------------------------------------------------------------------------HIKLATVLYHIGYIDIVYKI   [27]
Hypotrichomonas acosta                                       --------------------------------------------------------------------------------HIRFVRPLYHIGYIDIVYKI   [22]
Tritrichomonas foetus                                        --------------------------------------------------------------------------------HLKLAEPLFHIGYLDVVYKL   [27]
Dientamoeba fragilis                                         --------------------------------------------------------------------------------HINLAYPLFHFGYIDVVYKI   [26]
Monocercomonas sp. Ns-1PRR                                   --------------------------------------------------------------------------------HIELAEPVFHIGYIDIVYKI   [21]
Monocercomonas colubrorum                                    --------------------------------------------------------------------------------HIELAEPVFHIGYVDVVYKL   [20]
Pseudotrichomonas keilini NY0170                             -------------------------------------------------------------------------------------TPLFHVGFADKVYKL   [15]
Pentatrichomonas hominis                                        MKGIADDSSLIGSIDFGLFDPEEVKKFAACEITKPQNYENGKIVTGGLSDLRMGTQSPEYRCRTCGQDLQNCPGHFGYINLAEPFFHIGFSDKVYKI   [97]
Tetratrichomonas gallinarum turkey, Germany 4114-C5/05       -------------------------------------------------------------------------------------------GFHDKVYKL   [9]
Trichomonas tenax                                            --------------------------------------------------------------------------------YIKLAEPFFHVGFLKHVYKI   [26]
Trichomonas gallinae Coopers hawk 4 (US)                     --------------------------------------------------------------------------------YIKLAEPFFHVGFLKHVYKI   [27]
Trichomonas sp. RWG-2007-2 white winged dove 1200 (US)       --------------------------------------------------------------------------------YIRLAEPFFHVGFLPHCYKL   [21]
Trichomonas vaginalis C1:NIH                                 MANVRQNAIESCLVGSIDFGLFDPEKIEMFGACEITENQMMDKGEYVKGGLSDLRMGTQTRERLCDTCGRNQEDCPGHFGYIRLAEPFFHVGFLPHCYKL   [100]
                                                                                                                      Region A zinc finger

[                                                                      110       120       130       140       150       160       170       180       190       200]
[                                                                        .         .         .         .         .         .         .         .         .         .]
Trichomitus batrachorum                                      LQCVCPSCSRLLVGYADPHVQEAVLRYKGKKRLLAIHAIATKSSTRYCKHLEKKKSTKGVSGP----NQAQQSNPEEADYVKNDRFWKI----VTGSDDQ   [119]
Hypotrichomonas acosta                                       LQCVCSKCGRLLASYSQPELQSAVTHYHGRNRFLKVYEIIHGR-KAGCEHKKTKEMIKQQKAAQDQGAPAPAENAALADAAKNAEFWRR----VSNTLDE   [117]
Tritrichomonas foetus                                        MQCICHRCGRLKVNYAEPEFQRIIKNYHGKQRLIHMHEFCSSR--KKCDH-----------------VKVP--TPETPDVVKDISFWKELGVEIENEAQL   [106]
Dientamoeba fragilis                                         LNCVCHSCGRLLLSYGNPAVQRIVKNYQGKKRFDKIVEICRLS---SCEH-----------------EKRA-NDGAYDDIVKDKAFWDAIGI-VPEDGDY   [104]
Monocercomonas sp. Ns-1PRR                                   LQCVCHNCGRLLVSYAEPELQHIVLNYHGKQRLIKIHELLKSR--KSCCP-----------------SKGQNIDESCPDKVKDPAFWNELGV-FKDDDDY   [101]
Monocercomonas colubrorum                                    LQCVCHNCGRLLVSYSEPELQKIVLNYHGKQRLVRILELTKSR--HQCSH-----------------SKTPNGEDVHPDIVKNPAFWKELGV-VDEDGNY   [100]
Pseudotrichomonas keilini NY0170                             LQIVCHKCGRLLCDYGEIEIQKIVKHCQGKQRFLKIFEHIKSS-NHECKH-----------------KEST-KNKENPDFVEDKKFWRI----INGDSPH   [92]
Pentatrichomonas hominis                                     LQMVCHKCGRLLVSYSDPEVQYIVTHFKGKDRFTRIFEKISGK-THKCAH-----------------PTEKNKDPSQADEVVDEEFWNR----VNGGL-N   [174]
Tetratrichomonas gallinarum turkey, Germany 4114-C5/05       LQMVCHHCGRLNCNYSDPQLQQIVKNCKGKDRFNRVYELIGKKTGAKCKYAQKGEK-----------NPQEAQDEATYDMVSDDRFWEI----VNGGP-D   [93]
Trichomonas tenax                                            LQMVCHKCGRLNCSYGDPEIQYIATHYKGKQRFEKLFDYVADK-NKDCCD-----------------SPNA-KDQTNPDFVSDERFWEI----VNGDE-I   [102]
Trichomonas gallinae Coopers hawk 4 (US)                     LQMVCHKCGRLNCSYGDPEIQYIATHFKGKQRFDAIFNYIASS-TRDCSD-----------------SPAA-KDKESPDVVADERFWEI----VNGDA-I   [103]
Trichomonas sp. RWG-2007-1 ground dove 1 (US)                LQMVCHSCGRVLCNYSDPEIQFIVRHYKGKNRFLKLFEKISNKQCQKCSS-----------------GEDK-NSNKDMDCVSDDRFWEI----VNGDA-H   [78]
Trichomonas sp. RWG-2007-2 white winged dove 1200 (US)       LQMVCHKCGRVLCSYSDPEIQYIVTHYKGKNRFLKLFEKIASK-SGKCQH-----------------SPDL-KNPNEPDVCLDERFWEL----VNGDS-H   [97]
Trichomonas vaginalis C1:NIH                                 LQMVCHKCGRVLCSYSDPEIQYIVTHYKGKNRFLKLFEKIASK-SGKCQH-----------------SPDL-KNPNEPDVCLDERFWEL----VNGDS-H   [176]

[                                                                      210       220       230       240       250       260       270       280       290       300]
[                                                                        .         .         .         .         .         .         .         .         .         .]
                                                                                                                                RPB1AF2>
Trichomitus batrachorum                                      EKWKRESQPCGNAVPELTKENDGHIKKKDPARGEP--DLIESSKVLEILHNISEQDIRILGFDPQKCRPEWMMVKILPVPPPHVRPFIRQGG--HVTQDE   [215]
Hypotrichomonas acosta                                       SQWVRKSQPCGSKQNELEKNAQGRISRKDV-RGES--GIITAEVAYEILRQITDQDIRILGFDPIRCHPKWMILKVLPVPPLHVRPAVAMDG-VHKSQDD   [213]
Tritrichomonas foetus                                        SEDLRNKTPCSHDVPEISRDEDGNFRLKEHSGGGGGIDIISASQILEIFKQMSDYDILALGLDPKRSHPEWMICTVLPIPPPHVRPPVRAEG-SAPSQDD   [205]
Dientamoeba fragilis                                         SKDLRNIPTCGTIHYNVAREQDGTIRYKGKGNNE---E-FSAAAALDVFKLMKDCDIRVLGLDPERSHPKWMILTILPVPPPHVRPPVRVEG-LNPSPDD   [199]
Monocercomonas sp. Ns-1PRR                                   DEDFRNVQPCGQTVPEISKESDFTLKMKTPSKSSS--DILSAKSVLEILKHMNIYDIRVLGFNTERARPEWMISTILPVPPPHVRPAIVMDS-SRPSQDD   [198]
Monocercomonas colubrorum                                    DQDFRKMDPCGQMIPEITKDGDFTIKMKDPSRNAQ--DALSAKKVLEILKHLSPLDTKILGFDEEKAKPEWMICTILPVPPPHVRPAILMDG-SNMCQDD   [197]
Pseudotrichomonas keilini NY0170                             QECVRTKKPCNEKVPEVVQAKDFLVAYKDSRNYDG--DTISADHVFSIFENMSDQDIRILGFNPIRSHPKWLILKVLPVPPLAVRPQVEAPGQSSPSQDD   [190]
Pentatrichomonas hominis                                     GEHHRVKEPCNEAVPEVAMGKDFLVIYKDGSKTDE--DYIPAERVLNIFENISDQDLRIMGFDPKRSHPKWMILTVLPVPPLSVRPQVSSPGQAAPSQDD   [272]
Tetratrichomonas gallinarum turkey, Germany 4114-C5/05       GPHERLNRPCGKEIPGVEMGKDLMVKYKEDQSGV---DYPSAETVLRIFSSMTDQDVRILGFNVERSHPKWMILEVLPVPPLTVRPQVEAPGQSRPSQDD   [190]
Trichomonas tenax                                            SEHRRIKVPCKSKSVAIEQGKDFLIKYKDTSKTEE--DYLSAEYVLRIFENISDQDVRLMGFNVKRSHPKWMILKVLPVPPLAVRPQVVSPGQSVPSQDD   [200]
Trichomonas gallinae Coopers hawk4 (US)                      SEHRRMKVPCKSKPVAIEQGQDFLIKYKDTSKTEE--DYLSAEYVLRIFENISDQDVRLMGFNVKRSHPKWMILKVLPVPPLAVRPQVVSPGQSVPSQDD   [201]
Trichomonas sp. RWG-2007-1 ground dove 1 (US)                SDHVRIKKPCESTVPAVEQGKHFLIKYKDSSANED--DYIPAEKVLRIFENISDQDVRLLGFNVKRSHPKWLIIKILPVPPLAVRPQVVSPGQSVPSQDD   [176]
Trichomonas sp. RWG-2007-2 white winged dove 1200 (US)       SEHVRVKKPCNATVPAIEQGKDFLIKLKDVSKTEE--DYLSAEVVLRIFENISDQDVRLLGFNPKRSHPKWMILKILPVPPLAVRPQVVSPGQSAPSQDD   [195]
Trichomonas vaginalis C1:NIH                                 SEHVRVKKPCNAAVPAIEQGKDFLIKLKDVSKTEE--DYLSAEVVLRIFENISDQDVRLLGFNPKRSHPKWMILKILPVPPLAVRPQVVSPGQSAPSQDD   [274]

[                                                                      310       320       330       340       350       360       370       380       390       400]
[                                                                        .         .         .         .         .         .         .         .         .         .]
Trichomitus batrachorum                                      VSHQLGQIILLNKKVQEDITNGIPATKRQEDEFALQQCVTTYFINDKPSIDRATLKNGRPIKAISQRLKGKMGHIRGHLSGKRVNFSARSVISPDPSISI   [315]
Hypotrichomonas acosta                                       VTKKLSTIIMCNNSLENNINNGSANTVITEDLDTLQLHVTTYMVNDKPSIQRATMKNGRPFKAISQRLKGKSGHIRGHLSGKRVNFSSRSVISPDPSISI   [313]
Tritrichomonas foetus                                        VTHKLASIIQANNHLLKLKQEGAQQVALNENIELLQWHVTTYFINDKPSIKRATTKNGKPIKAISQRLKGKEGHIRGHLSGKRVDFSARSVISPDPSIRI   [305]
Dientamoeba fragilis                                         VTQHLGTILRCNNNLKKDKKGSNSQTTIKDSLGLLQWNVTTYFVNDKPSIKRAVTRNNKPIKSISQRLKGKEGHIRGHLNGKRVDFSARSVISPDPSIRI   [299]
Monocercomonas sp. Ns-1PRR                                   VTHKLATILQTNNHLKKLIQDNAQQVAQQDAIEMLQYHISTYLINDKPALQRATTKNGRPIKAISQRLKGKEGHIRGHLSGKRVNFSARSVISPDPSISI   [298]
Monocercomonas colubrorum                                    VTHKLASIVQVNNHLKKLIADNVQLVAKKEYIDLLQYHISTYFINDKPSLQRATTKNGRPIKAISQRLKGKEGHIRGHLSGKRVNFSARSVISPDPSISI   [297]
Pseudotrichomonas keilini NY0170                             VTHKLIDIIRINNSIADHTRNKGTETAIKQEKMLLQYHVTTYLINDKPSIARATTKNGRPLKVISMRLKGKEGHIRGHLSGKRVDFSARSVISPDPSISI   [290]
Pentatrichomonas hominis                                     VTHKLHSLLQTNEHLKLQLANGAPDTALAEDRQLLQWHISTYFINDKPSMDRAENKSHRPLKVISQRLKGKEGHIRGHLSGKRVNFSARSVISPDPSIEI   [372]
Tetratrichomonas gallinarum turkey, Germany 4114-C5/0        VTHKLVTIIQFNNRLAELKRQSATDTALKETRQFMQYHLTTYMINDKPSILRATTKNGRPLKVISQRLKGKEGHIRGHLSGKRDDFSARSVISPDPSISI   [290]
Trichomonas tenax                                            ITHKLSDIIKNNNHLAALRNDSSTDTAMNETRALLQYHITTYFINDKPSILRATTKNGRPLKVISQRLKGKEGHLRGHLSGKRDDFSARSVISPDPSISI   [300]
Trichomonas gallinae Coopers hawk4 (US)                      ITHKLVDIIKINNNLIALRNDSSTDTAMNDTRKLLQYHITTYFINDKPSILRATTKNGRPLKVISQRLKGKEGHLRGHLSGKRDDFSARSVISPDPSISI   [301]
Trichomonas sp. RWG-2007-1 ground dove 1 (US)                VTHKLSDIIQVNNKLTQLKNESSTDTAMKEVRQLLQYHITTYLCNDKPSIDRAQTKNGRPLKVISQRLKGKEGHLRGHLSGKRDDFSARSVISPDPSISI   [276]
Trichomonas sp. RWG-2007-2 white winged dove 1200 (US)       ITHKLSDIIICNKRLRAQRSESSTDTAMKETRTLLQYHITTYMINDKPSIERAVTKSGRPLKVISQRLKGKEGHLRGHLSGKRDDYSARSVISPDPSISI   [295]
Trichomonas vaginalis C1:NIH                                 ITHKLSDIIICNKRLRAQRSESSTDTAMKETRTLLQYHITTYMINDKPSIERAVTKSGRPLKVISQRLKGKEGHLRGHLSGKRDDYSARSVISPDTSISI   [374]
                                                                                                                         Region B, DNA binding
```

```
[                                                                     410       420       430       440       450       460       470       480       490       500]
[                                                                       .         .         .         .         .         .         .         .         .         .]
                                                              RPB1C2F>                                                                         TVRPB1DF>  RPB1C1F>
Trichomitus batrachorum                                         DQVGVPFELAKILTFPEVVNSLNIKDMQQLVYNGPDSQDGAKAVITPLGARFSLGDTQESTVQHLDYGSVVERHLRDNDIVIFNRQPSLHKMSMMGHYAK   [415]
Hypotrichomonas acosta                                          DQVGVPQEIARILTFPEVVTARNLTEMQRLVYNGNEAQEGANYVITPQGTRINLAVTQESTAMQLDHDSIVERHLRDDDIVIFNRQPSLHKMSMMGHRAR   [413]
Tritrichomonas foetus                                           DQVGVPQEIAKILTFPEVVTPRNREELTKLVINGPEELKGANYIITPQQMKIDLSHATERTAIHLDDLAIVERHLTDGDIVIFNRQPSLHKMSMMGHHAL   [405]
Dientamoeba fragilis                                            DQVGVPKHVAKILTFPEVVNPRNRDELYRLVQNGPDELQGANFVINPQGIRFSLSRTTERTTTHLDDGSIVERHLQDNDIVIFNRQPSLHKMSMMGHHAV   [399]
Monocercomonas spNs1PRR                                         DQVGVPLEIAKVLTFPEVVTPRNREYLQQLVYNGPDAQQGANYIITPQGIRINLAVTEERSALHLNDNAIVERHLRDDDIVIFNRQPSLHKMSMMGHRAY   [398]
Monocercomonas colubrorum                                       DQVGVPLEIAKVLTFPEVVTARNRDYLQKLVHNGPDHQDGANYIITPTGIRINLAVTEERSALHLADNAIVERHLREDDIVIFNRQPSLHKMSMMGHRAH   [397]
Pseudotrichomonas keilini NY0170                                DQVGVPKDLSKVLTFPEIVTKLNQKWLQGLVDLGPNEEGGANVIITDQGIKTDLEICKETCAQTISPGYIVERHIRDNDIVIFNRQPSLHKMSMMGHRAV   [390]
Pentatrichomonas hominis                                        DQVGVPKELAKILTFPEVVTTLNQKWLEGIVAKGPDAEGGANFIISDTGTKTDLQFCQKINTQNLSPGYTVERHLRDNDIVIFNRQPSLHKMSMMGHRAF   [472]
Tetratrichomonas gallinarum turkey, Germany 4114-C5/05          DQVGVPMELAKILTFPEIVTTTNQKWLESIVMKGPEDIGGANFVTSDHGVKTNLAMVNDLTSQTLSPGYIVERHIRDDDIVIFNRQPSLHKMSMMGHRAF   [390]
Trichomonas tenax                                               DQVGVPEDLAKILTFPEIVTTTNQKWLESIVMKGHDDIGGANYVTNDHGTKTDLAFCNDLSTIALSPGYVVDRHIRDNDIVIFNRQPSLHKMSMMGHRAL   [400]
Trichomonas gallinae Coopers hawk 4 (US)                        DQVGVPEDLAKILTFPEIVTTTNQKWLESIVMKGHDDIGGANYVTNDHGTKTDLAFCNDLSTIALSPGYIVDRHIRDNDIVIFNRQPSLHKMSMMGHRAL   [401]
Trichomonas sp. RWG-2007-1 ground dove 1 (US)                   DQVGVPEQLAKILTFPEVVTPINQKWLESIVQKGADSIGGANYVINDRGTRTDLSLCHDIMQVTLSNGYIVERHLRDDDIVIFNRQPSLHKMSMMGHRAL   [376]
Trichomonas sp. RWG-2007-2 white winged dove 1200 (US)          DQVGVPEQLAKILTFPEVVTPTNQKWLESIVMKGHDDLGGANFVINDHGTRTDLSCCTDLSTITLSPGYIVERHLRDDDIVIFNRQPSLHKMSMMGHRAL   [395]
Trichomonas vaginalis C1:NIH                                    DQVGVPEQLAKILTFPEVVTPTNQKWLESIVMKGHDDLGGANFVINDHGTRTDLSCCTDLSTITLSPGYIVERHLRDDDIVIFNRQPSLHKMSMMGHRAL   [474]
                                                                                                                                                 Region C

[                                                                     510       520       530       540       550       560       570       580       590       600]
[                                                                       .         .         .         .         .         .         .         .         .         .]
                                                                       RPB1DF>RPB1DF2><TVRPB1DR
Trichomitus batrachorum                                         LIRGQSFRLNLCVTTPYNADFDGDEMNLHVVQTQQARAEVKHIMAVPYQIITPQSNKPIIGLVQDCLVGCRLLSIRDTFLTRNELMNLMMWIMDTKPDLV   [515]
Hypotrichomonas acosta                                          IMPGQTFRLNLCVTTPYNADFDGDEMNMHVPQSQMARAEAKHIMLVPNQIISPQSACPIIGLVQDSLLGCRLLSLRDTFLTRNEITNLMMWIIDSN-DIV   [512]
Tritrichomonas foetus                                           ILPGSTFRLNLCVTTPYNADFDGDEMNLHVPQTQSARAEVKHIMLVPNQIITPQGNKPIIGLVQDSLLACRLLSLKSSFLNRNEMMNLMMWVRSKQ-NLV   [504]
Dientamoeba fragilis                                            IMRGSTFRLNLSVTTPYNADFDGDE---------------------------------------------------------------------------   [431]
Monocercomonas sp. Ns-1PRR                                      LMPGSTFRLNLCDTTPYNADFDGDEMNLHVPQSQTARAEVKHIMFVPNQIISPQANKPIIGLVQDALLACRLMTLRDTFLNRNQVMNLMMWIK-KR-DVV   [496]
Monocercomonas colubrorum                                       LMPGSTFRLNLCVTTPYNADFDGDEMNLHVPQSQSARAEVKHIMLVPNQIITPQSNKPIIGLVQDALLACRLMTLRDTFLTRNQVMNLMMWIK-KR-DVI   [495]
Monotrichomonas carabina                                        --------------------------NMHLPQSQAARAEIQHIMSVPYQIISPQGNSPIIGLVQDSLLGCRLMTFRDTFLEREEVMNLMMWIEGQE-DRP   [73]
Pseudotrichomonas keilini NY0170                                LMSGKTFRLNLCVTTPYNADFDGDE---------------------------------------------------------------------------   [422]
Pentatrichomonas hominis                                        LMSGKTFRLNLCVTTPYNADFDGDEMNLHVPQSQTARAEVRHIMAVPYQIISPQANKPIIGLVQDALLGSRLLSMKDTFLTRNQVMNLMMWLPNEK-DNI   [571]
Tetratrichomonas gallinarum turkey Germany 4114-C5/05           LISGRTFRLNLCDT--------------------------------------------------------------------------------------   [404]
Trichomonas tenax                                               LMPARTFRLNLCDTTPYNADFDGDEMNLHVPQSQTARAEVRHIMAVPKQIISPQANRPVIGLVQDALLGCRLLSKRDTFLTRNQVMNLMMWLPTNK-DTI   [499]
Trichomonas gallinae Coopers hawk 4 (US)                        LMPARTFRLNLCDTTPYNADFDGDEMNLHVPQSQAARAEVRHIMAVPKQIISPQANRPVIGLVQDALLGCRLLSKRDTFLTRNQVMNLMMWLPTNK-DTI   [500]
Trichomonas sp. RWG-2007-1 ground dove 1 (US)                   IMSGRTFRLNLCDTTPYNADFDGDEMNLHVPQSQTARTEVRHIMAVPKQIISPQANKPVIGLVQDALLGCRLLSKRDTFLTRNQVMNLMMWLPSTK-DTN   [475]
Trichomonas sp. RWG-2007-2 white winged dove 1200 (US)          IMSGRTFRLNLCDTTPYNADFDGDEMNLHVPQSQTARTEVRHIMAVPKQIISPQANKPVIGLVQDALLGCRLLSKRDTFLTRNQVMNLMMWLPTTK-DTI   [494]
Trichomonas vaginalis C1:NIH                                    IMSGRTFRLNPCDTTPYNADFDGDEMNLHVPQSQTARTEVRHIMAVPKQIISPQANKPVIGLVQDALLGCRLLSKRDTFLTRNQVMNLMMWLPTTK-DTI   [573]
                                                                         Region D, Mg++ binding

[                                                                     610       620       630       640       650       660       670       680       690       700]
[                                                                       .         .         .         .         .         .         .         .         .         .]
Trichomitus batrachorum                                         LPPPCIVAP---QELWSGKQVFSLFLPQINLDKFS--SPADDKGAD-KSWNSSDDVRVIIRDGHLLAGIIDSKTVAKSEGSLTHVVINSYSLDTAKAFLN   [609]
Hypotrichomonas acosta                                          LPPPCILKP---KELWSGKQVFSLFLPKINYDGFSVGADAKENTKEVNTWESPADLRVIIRNGNLLAGILDKKTVAKSAGSLNHVVINSYSIETARQFLN   [609]
Tritrichomonas foetus                                           LPPPCIIHPGNKVFLWSGKQVFSLFLPSINHDFVQ--KGAKEP----GSWIPVDDTRIIIRDGQLLAGILDKDTVAKSEKSLAHVVINSWDKNLARDFLS   [598]
Monocercomonas sp. Ns-1PRR                                      LPPPCILKP---QQLWSGKQVFSLFLPEINHDAFS--ADNKDD-----SWLPVDDKRVIIRDGKLLAGILDLKTVAKSEKSLVHVVINSWDKDIARDFLN   [586]
Monocercomonas colubrorum                                       LPPPCILKP---QQLWSGKQVFSLFLPEINHDGYS--SDVKDK-----SWLSYDDKRVIIRNGKLLAGILDSRTVARSEKSLVHVIINSWDKDIARDFLN   [585]
Monotrichomonas carabina                                        LPPPAILKP---KMLWTGKQIFSLFLPKINYDGWS--VTALDEEKKQNCWMSFNDTRVIIREGHLLAGILDKNTVARSEKSLVHIVINSYSTLIARDFLN   [168]
Pentatrichomonas hominis                                        LPPPCILKP---VQLWSGKQIFSLFMPRINLDAYS--ANVDEKRGDKKTMIPDNDCKVIIRNGELLAGIIDKKTVARGEQSLIHVVINSYDIKTAKDFLS   [666]
Trichomonas tenax                                               LPPPCILKP---VQLWSGKQVFSLFLPKISYDSYS--NGATDK--D-KNWIPKNDSRVIIRDGNLLAGILDKRSCARSEGSLTHVVINSYNTNIAKDFLN   [591]
Trichomonas gallinae Coopers hawk 4 (US)                        LPPPCILKP---VQLWSGKQVFSLFLPKINYDHFS--NGASDD--DKKSWMPANDTRVIIRDGHLLAGLLDKTSCARSEGSLTHVVINSYNTDIAKDFLN   [593]
Trichomonas sp. RWG-2007-1 ground dove 1 (US)                   LPPPCILKP---VQLWSGKQVFSLFLPKISHDSLS--QDATDA--D-KNSLPLNDRHVIIRDGNLLAGILDKRTVARSEGSLIHVVINSYNTNIAKDFLN   [567]
Trichomonas sp. RWG-2007-2 white winged dove 1200 (US)          LPPPCILKP---VQLWSGKQVFSLFLPKISHDSYS--QDANKM--D-QNSIPLADRHVIIRDGNLLAGILDKKTVARSEGSLIHVVINSYNTNIAKDFLN   [586]
Trichomonas vaginalis C1:NIH                                    LPPPCILKP---VQLWSGKQVFSLFLPKISHNSYS--QDANKM--D-QNSIPLADRHVIIRDGNLLAGILGKKTVARSEGSLIHVVINSYNTNIAKDFLN   [665]

[                                                                     710       720       730       740       750       760       770       780       790       800]
[                                                                       .         .         .         .         .         .         .         .         .         .]
Trichomitus batrachorum                                         QTQLVVNNWLENRGFSMGISTCVTTQEALDKVAGQLTELKAKI-ETIIEDAKHDRLEKMPGLTLTGTFEAKIMEVANDFTEKSSAVAIQAAPFWNALLQM   [708]
Hypotrichomonas acosta                                          QTQLIVNNWLENRGFSIGLSDCLAYEKTLDDVSDQIHQLKRNV-LDIIDQAKHGKLETPPGLTFMEGFESKINKLLNDLINDTGATVQKASRFWNSLMQM   [708]
Tritrichomonas foetus                                           ETQMVVNSWLESRGFSIGLIDAVATDKTNETVDQQIRDLESDV-KEIISRTQKGQLTIQPGMTLIESFEREVNTKLNKAIDACGSTVQKSSRFWNSLVQM   [697]
Monocercomonas sp. Ns-1PRR                                      QTQLVVNNWLELRGFSIGIIDCIASPRTLEKVSEQLGDLKKSV-DEITHSALNGTLKIQPGMTLKETFESEVNTRSNDFINKSGDVVKNDSRFWNSLMQM   [685]
Monocercomonas colubrorum                                       QTQLVVNNWLESRGFSIGIIDCIASEKTLIKVSEILDNLKEKV-DNITRSALSGTMTIQPGMTLKESFEQEVNSNSNDFINKSGAIVQNDARFWNSLMQM   [684]
Monotrichomonas carabina                                        VTQKVVNNWLESRGFSIGLSDCVTKKESLDQVHTTLSELKQSLEDEEIADAVSGRMEAEPGMTLKETFEFKVNAKLNKVLETAAGIVKKSISRENSLIQM   [268]
Pentatrichomonas hominis                                        QTQLIVNNWLEHRGFSIGLIDCVVPKFVTEEVDRALEELDRDV-NKEIRGAMSGQLEPAPGMTFMQTFEFNVNSKLNGFLGKSSDIVKAHIRRDNSLIEM   [765]
Trichomonas tenax                                               QTQLIVNNWLEYRGFSISLIDCVVPHFVLDKVEEGIMQVEENV-RETIRTAQSGKLDRRPGMTYIQSFEAEVNSQLNKVLSIAYKTITENIRPDNSFVEM   [690]
Trichomonas gallinae Coopers hawk 4 (US)                        QTQLIVNNWLEHRGFSIGLIDCVVPQSVLDQVEEKINEVEDNV-KGVIRTAQSGKLERRPGMTYIQSFEAEVNSALNQILGDAYKLIQKDIRRDNSFIEM   [692]
Trichomonas sp. RWG-2007-1 ground dove 1 (US)                   QTQLIVNNWLEHRGFSIGLIDCVVPSFVLEKVQDEITKVDNAV-KDTILKAKQGKLERMPGMSYMQSFETEVNNYLNEILNTTYKVINEKIRPDNSLKEM   [666]
Trichomonas sp. RWG-2007-2 white winged dove 1200 (US)          QTQLIVNNWLEHRGFSIGLIDCVVPDFVLQEVKHEIDEVESKV-QATIIKAQDGQLERMPGMSYMQSFETEVNNLTNEILSKTYKVINAKIRRDNSLSEM   [685]
Trichomonas vaginalis C1:NIH                                    QTQLIVNNWLEHRGFSIGLIDCVVPDFVLQEVKHEIDDVESKV-QATIIKAQDGQLERMPGMSYMQSFETEVNNLTNEILSKTYKVINAKIRGDNSLSEM   [764]
```

```
[                                                                            810       820       830       840       850       860       870       880       890       900]
[                                                                              .         .         .         .         .         .         .         .         .         .]
                                                                                                                                                   <RPB1FR
Trichomitus batrachorum                                    MQAGSKGSKVNIAQIIACVGQQSIEGERVRFGFKNRTLPHYVKDDFDLESRGFVEHSYIQGLTPQEFFFHSMGGRTGIIDTACKTADTGYIQRRLCKTME  [808]
Hypotrichomonas acosta                                     VAAGSKGSLINISQIIAVVGQQNVEGKRIRYGFKGRTLPHYVKDDYDLECRGFCEHSFIQGLTPQEFFFHAMGGRVGIIDTACKTSDTGYIQRRLCKSME  [808]
Tritrichomonas foetus                                      VKAGSKGSLLNISQIIATVGQQNIEGKRIRFGFKNRTLPHFTKDDFGLESRGFCRHCFLEGLNPPEFFFHAMAGREGIIDTACKTSDTGYIQRRLCKSME  [797]
Monocercomonas sp. Ns-1PRR                                 VAAGSKGSKINISQILACVGQQNVEGKRVPNGFKNRTLPHYFKDDVGLEAKGFCEHSYIQGLTPPEFFFHSMGGRTGIIDTACKTSDTGYIQRRLCKFME  [785]
Monocercomonas colubrorum                                  VAAGSKGSKINISQILACVGQQNVEGKRVPNGFKNRTLPHFFKDDMGLEAKGFCEHSYIQGLTPPEFFFHSMGGRTGIIDTACKTSDTGYIQRRLCKFME  [784]
Monotrichomonas carabina                                   VNAGSKGSNINISQILACVGQQNVEGKRIKYGFRGRTLPHYTKDDLGSESRGFVMNSYLIGLTPAEFFFHAMGGREGITDTACKTSDTGYIQRRLIKAME  [368]
Pentatrichomonas hominis                                   NMAGSKGADSNISQIIASVAQQNMEGKRVRFGFRMRTLPHYQKGMYGLIERGFCKHSYVEGLEPPEFIFHAMAGRTGIIDTACKTSDTGYIQRRLCKSME  [865]
Trichomonas tenax                                          LTAGSKGADFNITQVIGVVAQQNIEGKRVKFGFNGRTLPHYQKHEYGLVPRGFCKHSYIEGLTPPEFLFHAMGGRTGIIDTACKTSDTGYIQRRLVKSME  [790]
Trichomonas gallinae Coopers hawk 4 (US)                   LTAGSKGANFNVTQVIGVVAQQNIEGKRVKFGFNGRTLPHYQKHEYGLIPRGFCRHSYIEGLTPPEFLFHAMGGRTGIIDTACKTSDTGYIQRRLVKSME  [792]
Trichomonas sp. RWG-2007-1 ground dove 1 (US)              LSAGSKGADSNISQIIGVVGQQNMEGKRVKFGFSGRTLPHYQKHEYGLVPRGFCVNSYIAGLTPPEFLFHAMGGRTGIIDTACKTSDTGYIQRRLCKSME  [766]
Trichomonas sp. RWG-2007-2 white winged dove 1200 (US)     LSAGSKGADTNMSQIIGVVGQQNMEGKRVKFGFNGRTLPHFQKHEYGLVERGFCKNSYIAGLTPPEFLFHAMGGRTGIIDTACKTSDTGYIQRRLVKSME  [785]
Trichomonas vaginalis C1:NIH                               LSAGSKGADTNMSQIIGVVGQQNMEGKRVKFGFNGRTLPHFQKHEYGLVERGFCKNSYIAGLTPPEFLFHAMGGRTGIIDTACKTSDTGYIQRRLVKSME  [864]
                                                            Region E alpha amanitin sensitive/resistant; Translocation            Region F

[                                                                            910       920       930       940       950       960       970       980       990      1000]
[                                                                              .         .         .         .         .         .         .         .         .         .]
Trichomitus batrachorum                                    SHCVQYDGTVRDSMNNVVQFLYGGDGIDPVQIETQSLSLIALSDQEFDSMYKMNINDPVFGAGYLESSIISDLRKMEGTPTTTITGNFGGRSEEVEITRP  [908]
Hypotrichomonas acosta                                     SHCVMYDGTVRNSLNEIVQFIYGGDGLEPTKLETQKCPLIELSDRVFDERFVMEINDPTFGVGKIQRDIIENLRS--------------------DVRS-  [887]
Tritrichomonas foetus                                      SHCVMYDGTVRNSLNEVIQFIYGGDGLDPVGLETQNLTFMGDSDEDFDRKFRFDLTQAAFGQGIMDQEIVDELMS--------------------NPNHT  [877]
Monocercomonas sp. Ns-1PRR                                 SHCVQYDGTIRNSLNEVIQFLYGTDGMDPICLETQKIRLLSMDDNQFRNEYEFDLSLPTFGQGIMNADTIDEMNE--------------------NISAT  [865]
Monocercomonas colubrorum                                  SHCVQYDGTVRNSLNEIIQFVYGCDGMDPICLETQSIPLLEMDDNEFDREYEFDLSLPTFGQGIMRQEVIDGMND--------------------NISAT  [864]
Monotrichomonas carabina                                   SHQVAYDGTVRNSLNQIVQFIYGADGYDATSLETQNVRLVNMNDKAFRQNYELDIDNADFGIGFMEQHSIEELRN--------------------DPLA-  [447]
Pentatrichomonas hominis                                   SHHVAYDGTVRNSMNEVVQFIYGGDGLDATGLETQNLRLSVMGDREFSEEYEMEVDDATFGQNVMEQRIIDDVQN--------------------MQNR-  [944]
Trichomonas tenax                                          SHHVAYDGTVRNSLNEVVQFIYGGDGLDATGLETQFLNLVDLDDKKFDQTYHMNVDDPTFGSEAMDANILEEFLN--------------------TPGK-  [869]
Trichomonas gallinae Coopers hawk 4 (US)                   SHHVAYDGTVRNSLNEVVQFVYGGDGLDATGLETQRLTLVDCDDKTFDKTYHLSPEDPLFGTDCMDSNVLDEFLN--------------------TPGK-  [871]
Trichomonas sp. RWG-2007-1 ground dove 1 (US)              SHHVAYDGTVRNSQNEVVQFIYGGDGLDATGLETQRLGLVGLDDKAFNKLYHLNVDDPTFGQDAMDANVLEDFLR--------------------SMDK-  [845]
Trichomonas sp. RWG-2007-2 white winged dove 1200 (US)     SHHVAYDGTVRNSQNEIVQFIYGGDGLDATGLETQKLALVTLNDKEFMKKYHLASEDPTFGQVEMDDFVLDEFLK--------------------TPNK-  [864]
Trichomonas vaginalis C1:NIH                               SHHVAYDGTVRNSQNEIVQFIYGGDGLDATGLETQRLALVTLNDEDFMKKYHLATEDPTFGQVEMDDFVLDEFLK--------------------TPNK-  [943]

[                                                                           1010      1020      1030      1040      1050      1060      1070      1080      1090      1100]
[                                                                              .         .         .         .         .         .         .         .         .         .]
Trichomitus batrachorum                                    SAVLQEELDTLLHFRQILREEIFPNGNQKLYASVNLKRIIETAQRKYNINEYTSLSDLNPVLVVSEVKKLVSRLIIVQQNKDKIGREVQDNGTLLLRILI  [1008]
Hypotrichomonas acosta                                     QERLQAETKRLRDFRDLMRTEIFPNGDSVITLSMKVQRLIDTSKTVHDINEHTNISNLHPYFVIDEVDKLAKRLVVVPGD-DPIGREAQDNATLLMRIQL  [986]
Tritrichomonas foetus                                      KNRLDHEINRLLKFRQILREEIFTDASNKVWLPVNIRRLIESAQQSHHINPHSDKSNLNPLTVIQKVEELVDQLVIVKGE-DALSREAQDNATLLLRIHI  [976]
Monocercomonas sp. Ns-1PRR                                 RTILNRETERLLHFRKLLREEIFPSGEGSVYLPVNITRLLETAQQTHGINPHSDKSDLHPINVIESVENVINKLVVVPGD-DYISKEAQDNATLLLRILL  [964]
Monocercomonas colubrorum                                  KDVLQKEIARLKHFRRILREEVFPTATGSVYLPVNIGRLIQTAQQIHNINPHSDRSDLHPIHVIDSVEKLVSKLIVVKGD-DYIQKEAQDNATLLLRVLL  [963]
Monotrichomonas carabina                                   RDILNSEISNLSALRKKLREEVFTDAKNRVALPINISRLLETARMNYNINEYVSISDLRPLDVIKRVEEKIQSLIVVQGN-DRISRKAQENATLMLRAML  [546]
Pentatrichomonas hominis                                   HDVLNSEIKRLQDFRQLLQTEIFLKGESKVVLPVNITRIIQTSQQMQGINVHSSKSDLNPIDVIRRVDEKIRSLIVVKGT-DAIAVTAQENATLLLRIML  [1043]
Trichomonas tenax                                          EKKLQDEETRLKEFREILQTEIFPTGNKKVVLPINISRIIESSQRQFGINPHASTSDLNPLDVIAKVQDCVNSLTIVKGN-DSISLRAQENATLILKIML  [968]
Trichomonas gallinae Coopers hawk 4 (US)                   ENRLMHEENRLREFREILQTDIFPTGTTKVVLPVNIHRLIETSQRQCGINPRASISDLNPLDVISRVEECANSLTIVKGN-DSISRTAQENATLLLKIML  [970]
Trichomonas sp. RWG-2007-1 ground dove 1 (US)              DKKLQEEEHRLKEFREILQHEIFPDGSDTVVLPVNIARIIESSQRQFDINVHVSKSDLNPLDIIARVEDCVNSLIIVKGN-DTISRRAQDNATLLLRIML  [944]
Trichomonas sp. RWG-2007-2 white winged dove 1200 (US)     DKFLKEEENRLREYREILQKEIFPNGSNTVVLPVNIARIIESSQRQFDINVHVSKSDLNPLDIISRVDECVNSLIVVKGN-DNISRTEQENATLLLRIML  [963]
Trichomonas vaginalis C1:NIH                               DKFLKEEENRLREYREILQKEIFPNGSNTVVVPVNIARIIESSQRQFDINVHVSKSDLNPLDIISRVDECVNSLIVVKGN-DNISRTEQENATLLLRIML  [1042]

[                                                                           1110      1120      1130      1140      1150      1160      1170      1180      1190      1200]
[                                                                              .         .         .         .         .         .         .         .         .         .]
                                                                                               <RPB1GR2            <RPB1GR1
Trichomitus batrachorum                                    HATLASKPLIEKNRLSQKAFRSIIGEIEDRFYRTIVSPGEMVGTIAGQSIGEPATQ--------------------------------------------  [1070]
Hypotrichomonas acosta                                     YTMLASKPLILKERLSMDAFKWIIGEIEERFPETIVAPGEMVGTVAGQSIGEPSTQ--------------------------------------------  [1048]
Tritrichomonas foetus                                      FSNLASKPVIFQHRLTEQALIFVLGEIKTRFVQTIVSPGEMVGTIAGQSIGEPSTQMTLNTFHFAGVSAKNVTLGVPRLNEVMNLARVMKTPQVTVYLEP  [1076]
Monocercomonas sp. Ns-1PRR                                 YSNLAAKTLIFKYRLNEIAFKYVLGEVEVAFVKSIVSPGEMVGAIAGQSIGEPATQ--------------------------------------------  [1026]
Monocercomonas colubrorum                                  YSSLAAKCLIFKHRLSEDAFRYILGEGEHAFIGSIVSPGEMVGTIAGQSIGEPATQ--------------------------------------------  [1025]
Monotrichomonas carabina                                   YSNLSAKQLIFRHRLNSDAFEFVLGEITHLFKRSIVAPGEMVGTLAGQSI--------------------------------------------------  [596]
Pentatrichomonas hominis                                   YSNLSAKQIIFKHRLDEKAFEYVLGSISERFYRSIVSPGEMVGTIAGQSIGEPSTQMTLNTFHYAGISAKDVTLGVPRLNEIMNLAKQIKTPCVTVILDY  [1143]
Trichomonas tenax                                          YSNLASKQVIFTHRLNEQAFNWILGSIRDRFYRSIVAPGEMVGTIAGQSIGEPT----------------------------------------------  [1022]
Trichomonas gallinae Coopers hawk 4 (US)                   FSNLSAKQVIFEHRLNESAFSWILGAIHDRFYRSIVAPGEMVGTIAGQSIGEPTTQ--------------------------------------------  [1029]
Trichomonas sp. RWG-2007-1 ground dove 1 (US)              FSHLSAKQCIFKYRLNEQAFKYILGSVKDRFYRSICAPGEMVGTIAGQSIGEPS----------------------------------------------  [998]
Trichomonas sp. RWG-2007-2 white winged dove 1200 (US)     YSHLSAKQCIFEYRLNEQAFKYILGSIKDRFYRSIVAPGEMVGTIAGQSIGEPSTQ--------------------------------------------  [1026]
Trichomonas vaginalis C1:NIH                               YSHLSAKQCIFEYRLNEQAFKYILGSIKDRFYRSIVAPGEMVGTIAGQSIGEPSTQMTLNTFHFAGISAHDVTLGVPRLNELMNLAKHIRTPSVTVEFER  [1142]
                                                                                                      Region G, elongation, TFIIS binding

Tritrichomonas foetus                                      DSRG---------------------------DKDKAKDIQAELESSLKKLV-ARSEIYYDPSDMNSMIEE-DEWARYQ--GRED----SSNLSPWVLRLV  [1141]
Pentatrichomonas hominis                                   NSNNQDDDGSDMDDEEDRSVRQRQNKQELDAALAKDVRANVE-CAAFKKFVTKSEIFYDPEPADNTTIDEDREWIGIY-INNQLDFN-IDNYSPWVLRFE  [1240]
Trichomonas vaginalis C1:NIH                               DVAK-------------------------DEEIVKAVRAQIE-SASFKKFVIKAEIYYDPNPEETIIEPD-TKWINTRIRYGGVEDRELQDLAPWVLRFE  [1215]

Tritrichomonas foetus                                      LNQAALVDKNINPIEIVEKINILYANVYYAITNEGSSGDPIIRIRGLMNDKSFEDKDGRALQFAEQHLYDSLSLKGIEGINRAVMEETTRYEVDPESNAF  [1241]
Pentatrichomonas hominis                                   LDVKSLLASNVSLPEIQRAIENSYSSELFVSIFEGNMANAASSFPPVIRIRPTKSLVQKNGKTDESEFFLREIEQQLYDERAISIKGIPGIRRVKIDQKA  [1340]
Trichomonas vaginalis C1:NIH                               INRE-EMIASNIESISDIVQKIKGMYGDKFFVIGDDDARIIRMQARLKDEQAEFAMDVKFLRETEQQMYKSFTLKGIPGISRVKINSAAEKMFLNPETHG  [1314]

Tritrichomonas foetus                                      NTERKECKHKEWVLYTEGSALREVLNHPRVDCR------RSVSNNILETLEILGIEAARQSILI------------------------------------  [1299]
Pentatrichomonas hominis                                   KRKVLEEDH-HWVEKSDPQLITEGTALKAILGLDHIDKKLTYTNDINEIYDVLGIEAARNSLCNEMMMIMDNAGASLNRRHLDLLADTMTQYGRLYPVSR  [1439]
Trichomonas vaginalis C1:NIH                               FDKEKVSCL-YTEGTAFREILSLDHVDTV----------HTITNDIYQVCDVLGIEAARHSLYEMMLIMEKAGAALNRRHLDLLADTMTQYGVLYPVSR  [1403]
                                                                                                                                Region H
```
