# Supplementary material for: Phylogeny of Parasitic Parabasalia and Free-Living Relatives Inferred from Conventional Markers vs. Rpb1, a Single-Copy Gene
Source: PLoS One. 2011 Jun 9;6(6):e20774. doi: 10.1371/journal.pone.0020774 (PMC3111441; doi:10.1371/journal.pone.0020774)
Supplement: Figure S2 — Rooted eukaryotic Rpb1 phylogeny with constant sites removed recovers monophyletic Metamonada topology. New sequences from this study are indicated in bold type. This tree topology was calculated by RAxML 7.2.7 from 857 unambiguously aligned amino acids spanning conserved regions A to G of Rpb1, with constant sites removed. Thickened lines indicate the nodes supported by a Bayesian posterior probability of 1.00. Numbers at the nodes correspond to Bayesian posterior probabilities from the best post burn-in 1500 trees (chains run for 2×10^6^ generations), followed by percent bootstrap support ≥50% given by PhyML (100 replicates) and RAxML (1000 replicates). LnL = −56091.16, α = 1.33, pI = 0.0013. Scale bar represents 0.1 amino acid substitution per site. The alignment is provided in the Dataset S2. GenBank accession numbers, Joint Genome Institute or Broad Institute locus IDs are shown at the left for each taxon. (PDF) [file pone.0020774.s002.pdf]

Supporting Information for:

Phylogeny of parasitic Parabasalia and free-living relatives inferred from conventional markers *vs.* *Rpb1,* a single-copy gene

(*PLOS One,* 2011. doi:10.1371/journal.pone.0020774)

Shehre-Banoo Malik[1,2*§], Cynthia D. Brochu[2], Ivana Bilic[3], Jing Yuan[2], Michael Hess[3], John M. Logsdon Jr.[2], and Jane M. Carlton[1§]

[1] Department of Microbiology – Division of Medical Parasitology, New York University Langone Medical Center, New York NY, United States of America.

[2] Department of Biology – Roy J. Carver Center for Comparative Genomics, University of Iowa, Iowa City IA, United States of America.

[3] Department for Farm Animals and Veterinary Public Health – Clinic for Avian, Reptile and Fish Medicine, University of Veterinary Medicine, Vienna, Austria.

[*] current address:
Department of Biochemistry and Molecular Biology – Center for Comparative Genomics and Evolutionary Bioinformatics, Dalhousie University, Halifax NS, Canada.

[§] Corresponding Authors

E-mail addresses:
SBM: sbmalik@dal.ca
CDB: cindy-brochu@uiowa.edu
IB: Ivana.Bilic@vetmeduni.ac.at
JY: yuanjing2003@hotmail.com
MH: Michael.Hess@vetmeduni.ac.at
JML: john-logsdon@uiowa.edu
JMC: jane.carlton@nyumc.org

Figure S2: Rooted eukaryotic Rpb1 phylogeny with constant sites removed recovers monophyletic Metamonada topology.

New sequences from this study are indicated in bold type. This tree topology was calculated by RAxML 7.2.7 from 857 unambiguously aligned amino acids spanning conserved regions A to G of Rpb1, with constant sites removed. Thickened lines indicate the nodes supported by a Bayesian posterior probability of 1.00. Numbers at the nodes correspond to Bayesian posterior probabilities from the best post burn-in 1500 trees (chains run for $2x10^6$ generations), followed by percent bootstrap support $\geq$ 50% given by PhyML (100 replicates) and RAxML (1000 replicates). LnL = – 56091.16, α = 1.33, pI= 0.0013. Scale bar represents 0.1 amino acid substitution per site. The alignment is provided in the **Supporting Information Dataset S8**. GenBank accession numbers, Joint Genome Institute or Broad Institute locus IDs are shown at the left for each taxon.

Figure S2

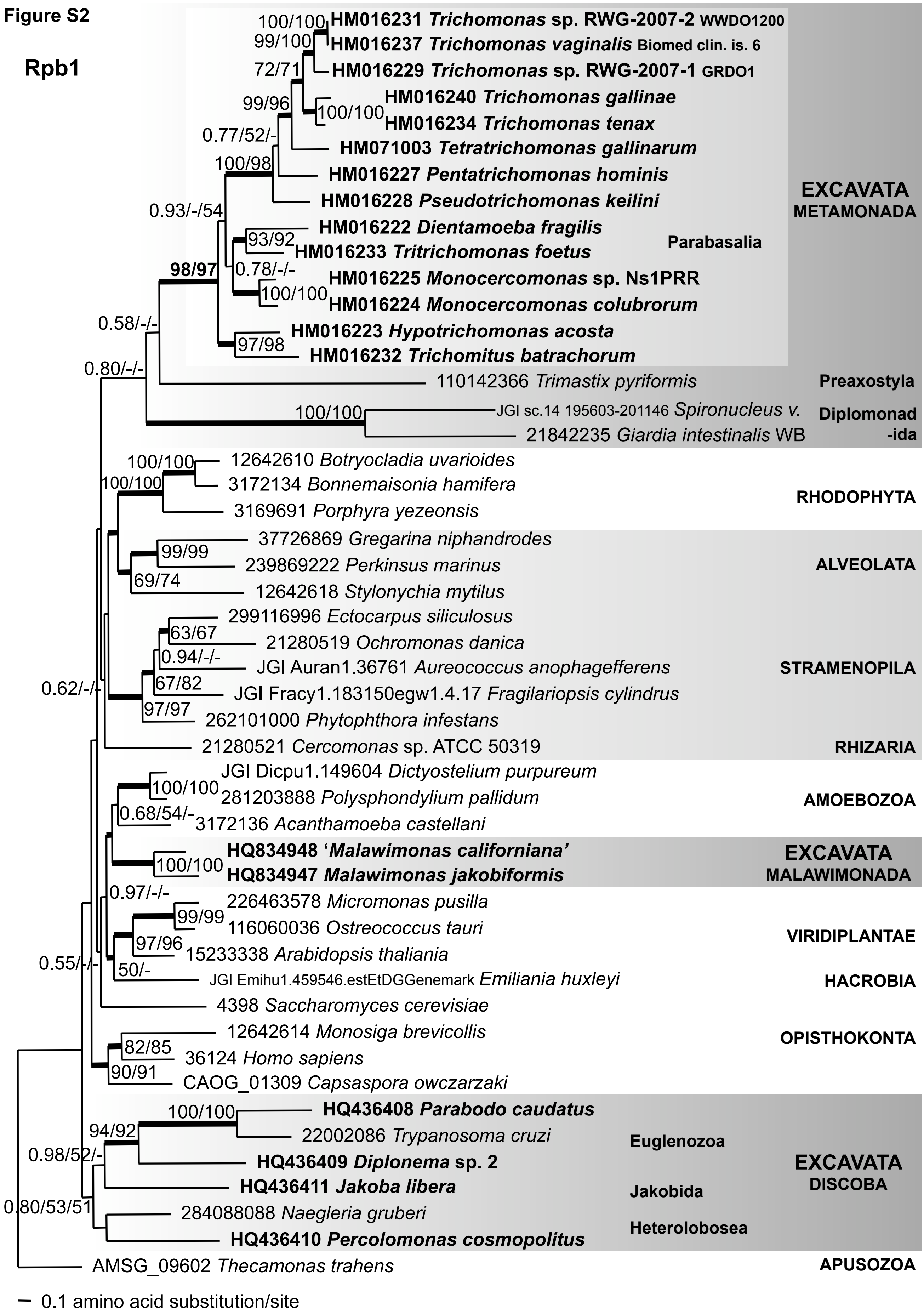
Rpb1
100/100 HM016231 Trichomonas sp. RWG-2007-2 WWDO1200
99/100 HM016237 Trichomonas vaginalis Biomed clin. is. 6
72/71 HM016229 Trichomonas sp. RWG-2007-1 GRDO1
99/96 HM016240 Trichomonas gallinae
100/100 HM016234 Trichomonas tenax
0.77/52/- HM071003 Tetratrichomonas gallinarum
100/98 HM016227 Pentatrichomonas hominis
HM016228 Pseudotrichomonas keilini
0.93/-/54 HM016222 Dientamoeba fragilis
93/92 HM016233 Tritrichomonas foetus
98/97 0.78/-/- HM016225 Monocercomonas sp. Ns1PRR
100/100 HM016224 Monocercomonas colubrorum
0.58/-/- HM016223 Hypotrichomonas acosta
97/98 HM016232 Trichomitus batrachorum
Parabasalia
EXCAVATA
METAMONADA
0.80/-/- 110142366 Trimastix pyriformis
Preaxostyla
100/100 JGI sc.14 195603-201146 Spironucleus v.
21842235 Giardia intestinalis WB
Diplomonad -ida
100/100 12642610 Botryocladia uvarioides
100/100 3172134 Bonnemaisonia hamifera
3169691 Porphyra yezeonsis
RHODOPHYTA
37726869 Gregarina niphandrodes
99/99 239869222 Perkinsus marinus
69/74 12642618 Stylonychia mytilus
ALVEOLATA
299116996 Ectocarpus siliculosus
63/67 21280519 Ochromonas danica
0.94/-/- JGI Auran1.36761 Aureococcus anophagefferens
67/82 JGI Fracy1.183150egw1.4.17 Fragilariopsis cylindrus
0.62/-/- 97/97 262101000 Phytophthora infestans
STRAMENOPILA
21280521 Cercomonas sp. ATCC 50319
RHIZARIA
JGI Dicpu1.149604 Dictyostelium purpureum
100/100 281203888 Polysphondylium pallidum
0.68/54/- 3172136 Acanthamoeba castellani
AMOEBOZOA
HQ834948 'Malawimonas californiana'
100/100 HQ834947 Malawimonas jakobiformis
EXCAVATA
MALAWIMONADA
0.97/-/- 226463578 Micromonas pusilla
99/99 116060036 Ostreococcus tauri
97/96 15233338 Arabidopsis thaliania
VIRIDIPLANTAE
0.55/-/- 50/- JGI Emihu1.459546.estEtDGGenemark Emiliania huxleyi
HACROBIA
4398 Saccharomyces cerevisiae
12642614 Monosiga brevicollis
82/85 36124 Homo sapiens
90/91 CAOG_01309 Capsaspora owczarzaki
OPISTHOKONTA
100/100 HQ436408 Parabodo caudatus
94/92 22002086 Trypanosoma cruzi
Euglenozoa
0.98/52/- HQ436409 Diplonema sp. 2
HQ436411 Jakoba libera
Jakobida
EXCAVATA
DISCOBA
0.80/53/51 284088088 Naegleria gruberi
HQ436410 Percolomonas cosmopolitus
Heterolobosea
AMSG_09602 Thecamonas trahens
APUSOZOA
0.1 amino acid substitution/site
