# Supplementary material for: Phylogeny of Parasitic Parabasalia and Free-Living Relatives Inferred from Conventional Markers vs. Rpb1, a Single-Copy Gene
Source: PLoS One. 2011 Jun 9;6(6):e20774. doi: 10.1371/journal.pone.0020774 (PMC3111441; doi:10.1371/journal.pone.0020774)
Supplement: Figure S3 — GAPDH phylogeny rooted with Preaxostyla and Bacteria does not resolve interrelationships of six parabasalid groups. This consensus topology of the 9500 best trees calculated by Bayesian inference was constructed from 254 unambiguously aligned amino acids, with constant sites removed. LnL = −8779.10, α = 1.52 (1.25<α<1.83), pI = 0.0039 (0.000094<pI<0.014). Scale bar represents 0.1 amino acid substitution per site. Thickened lines indicate the nodes supported by a Bayesian posterior probability of 1.00. Numbers at the nodes correspond to Bayesian posterior probabilities, followed by percent bootstrap support ≥50% given by PhyML and RAxML (1000 replicates each). Data generated in this study is highlighted by bold type. The alignment is provided in the Dataset S3. Genbank accession numbers are shown at the left for each taxon. (PDF) [file pone.0020774.s003.pdf]

Supporting Information for:

Phylogeny of parasitic Parabasalia and free-living relatives inferred from conventional markers *vs.* *Rpb1,* a single-copy gene

(*PLOS One,* 2011. doi:10.1371/journal.pone.0020774)

Shehre-Banoo Malik[1,2*§], Cynthia D. Brochu[2], Ivana Bilic[3], Jing Yuan[2], Michael Hess[3], John M. Logsdon Jr.[2], and Jane M. Carlton[1§]

[1] Department of Microbiology – Division of Medical Parasitology, New York University Langone Medical Center, New York NY, United States of America.

[2] Department of Biology – Roy J. Carver Center for Comparative Genomics, University of Iowa, Iowa City IA, United States of America.

[3] Department for Farm Animals and Veterinary Public Health – Clinic for Avian, Reptile and Fish Medicine, University of Veterinary Medicine, Vienna, Austria.

[*] current address:
Department of Biochemistry and Molecular Biology – Center for Comparative Genomics and Evolutionary Bioinformatics, Dalhousie University, Halifax NS, Canada.

[§] Corresponding Authors

E-mail addresses:
SBM: sbmalik@dal.ca
CDB: cindy-brochu@uiowa.edu
IB: Ivana.Bilic@vetmeduni.ac.at
JY: yuanjing2003@hotmail.com
MH: Michael.Hess@vetmeduni.ac.at
JML: john-logsdon@uiowa.edu
JMC: jane.carlton@nyumc.org

Figure S3: GAPDH phylogeny rooted with Preaxostyla and Bacteria does not resolve interrelationships of six parabasalid groups.
This consensus topology of the 9500 best trees calculated by Bayesian inference was constructed from 254 unambiguously aligned amino acids, with constant sites removed. LnL = – 8779.10, $\alpha = 1.52$ ($1.25 < \alpha < 1.83$), pI= 0.0039 ($0.000094 <$ pI < 0.014). Scale bar represents 0.1 amino acid substitution per site. Thickened lines indicate the nodes supported by a Bayesian posterior probability of 1.00. Numbers at the nodes correspond to Bayesian posterior probabilities, followed by percent bootstrap support $\geq$ 50% given by PhyML and RAxML (1000 replicates each). Data generated in this study is highlighted by bold type. The alignment is provided in the **Supporting Information Dataset S2.** Genbank accession numbers are shown at the left for each taxon.

Figure S3

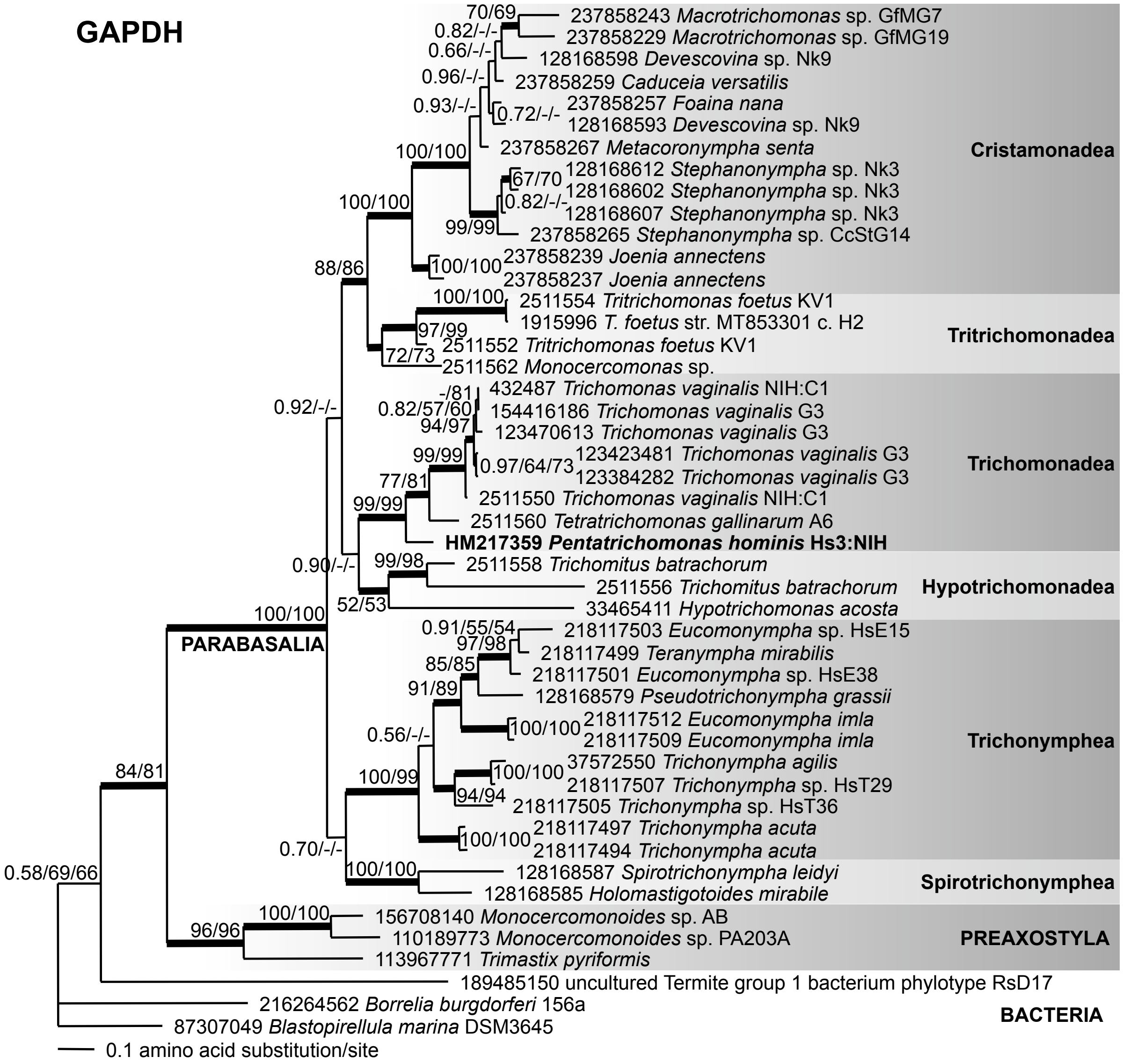
GAPDH
70/69
0.82/-/-
0.66/-/-
0.96/-/-
0.93/-/-
0.72/-/-
237858243 *Macrotrichomonas* sp. GfMG7
237858229 *Macrotrichomonas* sp. GfMG19
128168598 *Devescovina* sp. Nk9
237858259 *Caduceia versatilis*
237858257 *Foaina nana*
128168593 *Devescovina* sp. Nk9
237858267 *Metacoronympha senta*
100/100
67/70
0.82/-/-
99/99
128168612 *Stephanonympha* sp. Nk3
128168602 *Stephanonympha* sp. Nk3
128168607 *Stephanonympha* sp. Nk3
237858265 *Stephanonympha* sp. CcStG14
100/100
88/86
100/100
237858239 *Joenia annectens*
237858237 *Joenia annectens*
Cristamonadea
100/100
97/99
72/73
2511554 *Tritrichomonas foetus* KV1
1915996 *T. foetus* str. MT853301 c. H2
2511552 *Tritrichomonas foetus* KV1
2511562 *Monocercomonas* sp.
Tritrichomonadea
0.92/-/-
-/81
0.82/57/60
94/97
99/99
0.97/64/73
77/81
99/99
432487 *Trichomonas vaginalis* NIH:C1
154416186 *Trichomonas vaginalis* G3
123470613 *Trichomonas vaginalis* G3
123423481 *Trichomonas vaginalis* G3
123384282 *Trichomonas vaginalis* G3
2511550 *Trichomonas vaginalis* NIH:C1
2511560 *Tetratrichomonas gallinarum* A6
HM217359 *Pentatrichomonas hominis* Hs3:NIH
Trichomonadea
0.90/-/-
99/98
52/53
2511558 *Trichomitus batrachorum*
2511556 *Trichomitus batrachorum*
33465411 *Hypotrichomonas acosta*
Hypotrichomonadea
100/100
PARABASALIA
0.91/55/54
97/98
85/85
91/89
0.56/-/-
100/100
100/99
100/100
94/94
100/100
218117503 *Eucomonympha* sp. HsE15
218117499 *Teranympha mirabilis*
218117501 *Eucomonympha* sp. HsE38
128168579 *Pseudotrichonympha grassii*
218117512 *Eucomonympha imla*
218117509 *Eucomonympha imla*
37572550 *Trichonympha agilis*
218117507 *Trichonympha* sp. HsT29
218117505 *Trichonympha* sp. HsT36
218117497 *Trichonympha acuta*
218117494 *Trichonympha acuta*
Trichonymphea
84/81
0.70/-/-
100/100
128168587 *Spirotrichonympha leidyi*
128168585 *Holomastigotoides mirabile*
Spirotrichonymphea
0.58/69/66
96/96
100/100
156708140 *Monocercomonoides* sp. AB
110189773 *Monocercomonoides* sp. PA203A
113967771 *Trimastix pyriformis*
PREAXOSTYLA
189485150 uncultured Termite group 1 bacterium phylotype RsD17
216264562 *Borrelia burgdorferi* 156a
87307049 *Blastopirellula marina* DSM3645
BACTERIA
0.1 amino acid substitution/site
