# Supplementary material for: Phylogeny of Parasitic Parabasalia and Free-Living Relatives Inferred from Conventional Markers vs. Rpb1, a Single-Copy Gene
Source: PLoS One. 2011 Jun 9;6(6):e20774. doi: 10.1371/journal.pone.0020774 (PMC3111441; doi:10.1371/journal.pone.0020774)
Supplement: Table S1 — Primers used to amplify and sequence fragments of parabasalid Rpb1 genes. Primers are listed from 5′ to 3′ positions within the gene. (PDF) [file pone.0020774.s004.pdf]

Supporting Information for:

Phylogeny of parasitic Parabasalia and free-living relatives inferred from conventional markers *vs. Rpb1,* a single-copy gene

(*PLOS One,* 2011. doi:10.1371/journal.pone.0020774)

Shehre-Banoo Malik[1,2*§], Cynthia D. Brochu[2], Ivana Bilic[3], Jing Yuan[2], Michael Hess[3], John M. Logsdon Jr.[2], and Jane M. Carlton[1§]

[1] Department of Microbiology – Division of Medical Parasitology, New York University Langone Medical Center, New York NY, United States of America.

[2] Department of Biology – Roy J. Carver Center for Comparative Genomics, University of Iowa, Iowa City IA, United States of America.

[3] Department for Farm Animals and Veterinary Public Health – Clinic for Avian, Reptile and Fish Medicine, University of Veterinary Medicine, Vienna, Austria.

[*] current address:
Department of Biochemistry and Molecular Biology – Center for Comparative Genomics and Evolutionary Bioinformatics, Dalhousie University, Halifax NS, Canada.

[§] Corresponding Authors

E-mail addresses:
SBM: sbmalik@dal.ca
CDB: cindy-brochu@uiowa.edu
IB: Ivana.Bilic@vetmeduni.ac.at
JY: yuanjing2003@hotmail.com
MH: Michael.Hess@vetmeduni.ac.at
JML: john-logsdon@uiowa.edu
JMC: jane.carlton@nyumc.org

Table S1: Primers used to amplify and sequence fragments of parabasalid *Rpb1* genes, listed from 5' to 3' positions within the gene.

	**Forward primer (5' – 3')**	**Reverse primer (5' – 3')**
**General degenerate oligos**		
Rpb1AF1 (ECPGHFG) [56]	GAGTGTCCAGGNCAYTTYGG	
Rpb1AF2 (SHPKWMI)	TCNCATCCARARTGGATGAT	
Rpb1C2F (IDQVGVP)	ATMGAYCARGTNGGWGTTCC	
Rpb1C1F (KMSMMGH)	AARATGTCYATGATGGGNCAC	
Rpb1DF (TTPYNA)	ACWACWCCWTAYAAYGC	
Rpb1DF2 (PYNADFDGDEM)	CCGTACAATGCAGAYTTYGAYGGNGAYGARATG	
Rpb1FR (FHAMGG)		CCAGCCATNGCRTGRAA
Rpb1GR2 (PGEMVGT)		GTGCCGACCATYTCNCCNGG
Rpb1GR1 (MTLNTFH) [56]		GTGGAACGTGTTNARNGTCAT
Trichomonas vaginalis		
TrichomonRpb1ASR1		ATGTATTGAATTTCTGGATC
TvRpb1iF	GAAGATTATTTATCCGCCG	
TvRpb1iR		GGGAGTATGAGTTGTGGG
TvRpb1DF (IFNRQP)	CATTTTCAACCGTCAGCC	
**TvRpb1DR (MNLHVPQ)**		TGTGGAACGTGAAGGTTCAT
TvB7Rpb1SFD2	GCGCGATACATTCCTTAC	
TvRpb1SF1	ACTCCATTCCTCTTGCGG	
TvRpb1SF2	GGTAACCTCCTCGCTGG	
TvRpb1SF3	GGTCTCATCGACTGCG	
TvRpb1GSF4	TGATAACATCTCTCGTAC	
TvRpb1SR1		CATCGAGAACGAAATCGTCC
Trichomonas tenax		
TtxRpb1SF1	GGATGTCAGACTCATGGGC	
TtxRpb1SF2	GCTGATTTCAACATCACACAGG	
TtxRpb1SF3		
TtxRpb1SF4	GATCAATCGTCGCTCCAGG	
TtxRpb1SR1		CCGTTTGAATATGAGTCATATG
TtxRpb1SR3 (GHLRGH)		GTGTCCTCTCAAGTGACC
TtxRpb1SR4		GGGTGAATCACAGCAGTCC
***Trichomonas* sp. RWG-2007-1 GRDO1**		
Tg393Rpb1ASF2	TCCAACAAACAATGCC	
Tg393Rpb1CSR1		CTGTTCTTGTACCACGATC
Tg393Rpb1DSF1	CTGTTGCTAGATCAGAAGG	
Tg393Rpb1ESF3	ATTCGGCTTTAGTGGTAG	
Tg393Rpb1GSF4	CAAGACGTGCTCAAGATAATGC	
Trichomonas gallinae		
Tg394Rpb1BSF1	GGCCAAAGCGTCCCATCACAGG	
Tg394Rpb1ESR1		GCACCCTTCGAACCGGCAGTAAGC
Tg394Rpb1GSF2	GCGCAGGAGAACGCCACGC	
Tg8855C306Rpb1R4		GGGACTTTCATACGTCTGTG
Tg8855C306Rpb1F2	GCTGAAAGTTATCTCACAGCGTC	

Tg8855C306Rpb1R3		GTCTTCGACTTCGTTAATCTTCTC
Tg8855C306Rpb1R2		TGAGGAACTCGTCGAGTACG
Tg8855C306Rpb1F3	AGAAACTCTCTCAACGAAGTTGTC	
Tg8855C306Rpb1F4	CGCGTGCTTCAATTTCCGAC	
Pentatrichomonas hominis		
PhRpb1SF1 (DTGTKTD)	CAGATACGGGAACGAAGACCG	
PhRpb1SF2	GCTGTGGTCAGGTAAGC	
PhRpb1SF3	GGCGCACATCCGCCGCG	
PhRpb1SR1 (TYFIND)		ATCATTGATAAAGTATGT
PhRpb1SR2		CCTCGTCAGCTTGAGATGGG
PhRpb1SR3		CGACTTCCATCTCGTACTCCTC
PhRpb1GSF1	CAAGCACAGGCTCGACG	
Pseudotrichomonas keilini		
PkRpb1ASR1		GCATTCATGATTTGACGAC
PkRpb1BSF1	GATCGCATCCCAAGTGG	
PkRpb1DSF1	ATAGACAGCCATCACTCC	
PkRpb1DSF2	GAGCTGTATTAATGAGTGG	
Tritrichomonas foetus		
TfRpb1SF1 (SHPEWM)	TCGCATCCAGAATGGATG	
TfRpb1SF2 (TDGDIV)	ACCGATGGTGATATTGTT	
TfRpb1SF3	GGATACCTGTTGATGATACACG	
TfRpb1SF4	GCACTTTCTAGAGAAGC	
TfRpb1SR3		GTGACCACGAATGTGTCC
TfRpb1SR4		GGTTAGGATTACTCATTAATTC
Dientamoeba fragilis		
DfRpb1SF1 (PVPPPH)	CCAGTTCCACCACCACACG	
DfRpb1SF2 (SIVERH)	CTATCGTTGAGCGTCACC	
DfRpb1DSF3	GTCACCATGCTGTGATTATGAG	
DfRpb1ASR1		GCTCGCATGATGATAAGCGGC
DfRpb1SR1 (DDGSIV)		CGATAGAGCCATCATCC
Trichomitus batrachorum		
TbRpb1ASF1	GGACCCTGCGCGTGGAGAACCCG	
TbRpb1ASR1		TGCATGAATGGCCAGAAGAC
TbRpb1CSR2		CGATGGAGATCGAAGGATCGGG
TbRpb1DSF2	ATGTCCATGATGGGGCACTATGC	
TbRpb1GSR3		CGATGATACGCTTCAGG
TbRpb1GSF4	TTCATGCCACGCTCGCCTC	
TbRpb1DSR4		GTGTCCATAATCCACAT
TbRpb1DSF5	TGGACACAAAGCCTGACC	
TbRpb1ESF6	CGCTCACCGGTACATTTGAAGCG	
TbRpb1FSR4		CGCGAACGGTTCCGTCGTATTGG
Hypotrichomonas acosta		
HaRpb1ASR1		GGTAATGTGTGACAGCGC
HaRpb1AF1	ATGCGGTCGTTTACTTGC	

HaRpb1CSR1		CCTGTGGTGTAATGACATAG
HaRpb1DSR1		CCACATCATCAAGTTTG
HaRpb1DSF1	CAATCGTCAACCATCTCTCC	
HaRpb1FF1	CTCCTCCAGGTCTCACTTTCATGG	
HaRpb1FR1		TGCACAGTGGCACCAGT
HaRpb1GSF1	GCCCAAGACAATGCAACTC	
Monocercomonas colubrorum		
McRpb1ASR1		GATACAAGTAAACGGCCAC
McRpb1BSF1	GGAGAAGATGTTCATCCTG	
McRpb1DSR1		CTGGTCCATTATGGACAAGC
McRpb1DSF1	GTAGAACGCCATCTCCGAG	
McRpb1ESF1	GCCTGAAATTAACCATGATGG	
McRpb1GSF1	GAAGCTCAAGATAATGCAAC	
McRpb1GSR1		CTGATCTATCAGAATGTGG
***Monocercomonas* sp.**		
MspRpb1ASF1	CAAAGTTAAAGATCCCGC	
MspRpb1ASR1		ATGAGCCTCTGCTTTCCATG
MspRpb1DSR1		GCTATCTCAAGTGGCACACC
MspRpb1DF1	AAAATGTCTATGATGGGTC	
MspRpb1ESF1	GCGCGATGTCGTTCTCCCACC	
MspRpb1FSR1		GTGAGTTACGAATAGTTCC
MspRpb1GSR1		CCTTGGAAATGTAATCATC
MspRpb1GSF1	GATGATTACATTTCCAAGG	
Monotrichomonas carabina		
MtRpb1DR1		GAACCAAGCCGATGATTGG
