# Supplementary material for: Phylogeny of Parasitic Parabasalia and Free-Living Relatives Inferred from Conventional Markers vs. Rpb1, a Single-Copy Gene
Source: PLoS One. 2011 Jun 9;6(6):e20774. doi: 10.1371/journal.pone.0020774 (PMC3111441; doi:10.1371/journal.pone.0020774)
Supplement: Table S2 — Primers used to amplify and sequence fragments of Discoba Rpb1 genes. Primers for Parabodo caudatus, Diplonema sp. 2, Percolomonas cosmopolitus, Jakoba libera and Malawimonas are listed from 5′ to 3′ positions within the gene. (PDF) [file pone.0020774.s005.pdf]

Supporting Information for:

Phylogeny of parasitic Parabasalia and free-living relatives inferred from conventional markers *vs.* *Rpb1,* a single-copy gene

(*PLOS One,* 2011. doi:10.1371/journal.pone.0020774)

Shehre-Banoo Malik[1,2*§], Cynthia D. Brochu[2], Ivana Bilic[3], Jing Yuan[2], Michael Hess[3], John M. Logsdon Jr.[2], and Jane M. Carlton[1§]

[1] Department of Microbiology – Division of Medical Parasitology, New York University Langone Medical Center, New York NY, United States of America.

[2] Department of Biology – Roy J. Carver Center for Comparative Genomics, University of Iowa, Iowa City IA, United States of America.

[3] Department for Farm Animals and Veterinary Public Health – Clinic for Avian, Reptile and Fish Medicine, University of Veterinary Medicine, Vienna, Austria.

[*] current address:
Department of Biochemistry and Molecular Biology – Center for Comparative Genomics and Evolutionary Bioinformatics, Dalhousie University, Halifax NS, Canada.

[§] Corresponding Authors

E-mail addresses:
SBM: sbmalik@dal.ca
CDB: cindy-brochu@uiowa.edu
IB: Ivana.Bilic@vetmeduni.ac.at
JY: yuanjing2003@hotmail.com
MH: Michael.Hess@vetmeduni.ac.at
JML: john-logsdon@uiowa.edu
JMC: jane.carlton@nyumc.org

Table S2: Primers used to amplify and sequence fragments of *Parabodo caudatus, Diplonema* sp. 2*, Percolomonas cosmopolitus*, *Jakoba libera* and *Malawimonas Rpb1* genes, listed from 5' to 3' positions within the gene.

	Forward primer (5' – 3')	Reverse primer (5' – 3')
**General degenerate oligos**		
Rpb1AF1 (ECPGHFG) [56]	GAGTGTCCAGGNCAYTTYGG	
Rpb1BF2 (RGNLMGK)	AGAGGTAACTTRATGCNAA	
Rpb1DF3 (PYNADFG)	CCGTACAATGCAGAYTTYGAYGG	
Rpb1DF4 (ADFDGDEM)	GCAGACTTCGATGGNGAYGARATG	
Rpb1EF5 (P(L/I)WTGKQ)	CCACTGTGGACNGGNAARCA	
Rpb1DR7 (PYNADFD)		GTCGAAGTCTGCRTTRTANGG
Rpb1FR5 (FH(A/T)M(G/A)GRE)		ACGACCTGCCATNGYRTGRAA
Rpb1GR2 (MTLNTFH) [56]		GTGGAACGTGTTNARNGTCAT
Rpb1GR9 (VTLGVPR)		TCTAGGCACTCCGARNGTNAC
Parabodo caudatus		
37BodoRpb1R1		CAAAGTACTTGTCGAGGAAGAC
***Diplonema* sp. 2**		
34Rpb1F1	CTACACAGAATGAGTATGATGGG	
34Rpb1F2	TGTTCCTTAATCAAACTAGATCC	
41Rpb1R1		GTTGACCGCTTTGTGAGCAGATG
Percolomonas cosmopolitus		
PerRpb1F1	GGTCAACAGAATATTGAAGG	
PerRpb1F2	AGACGATGGTTGTTGGTGG	
PerRpb1R1		AGTGCATCTTGCACGATAGC
PerRpb1R2		GCTCACCACGACTGATGACG
PerRpb1R3		TCAACGGATGTCCCATCTTTGTG
Jakoba libera		
28Rpb1F0	TTGCTGAACCCGCTCATGTCA	
28Rpb1F1	CATTTCCGCCAGGAGAACTATG	
28Rpb1F2	GATTGGTGAAAGCCATGGAGGA	
28Rpb1SF3	GTCGGTTAGTTTGTTGACT	
28Rpb1F5	GTCCATGTCCCAGAATGCA	
28Rpb1SF6	GTATGCGTTGTTCCAAACT	
28Rpb1SF7	GACATTGACCATGGGT	
28Rpb1R0		GACCGTCAGAAGCCAGTAGTTG
28Rpb1R1		GACACCAGAGTGTTCTCCGAG
28Rpb1R2		CAACTCTCCTCGACCTGAACTA
28Rpb1SR3		CAGATATGCAGCACACATG
28Rpb1SR4		CAACCATACATGCAAACGAG
28Rpb1R5		ACGCACCTCTTGATGTTGA
28Rpb1R6		CATTGCATGTGGAGTACCAAG
28Rpb1R8		CTCGAAGAGGTAGGTGGCATT
28Rpb1R9		GAAGCTGACCCTTTCCAAG
Malawimonas jakobiformis		
MjakRpb1SF1	GATGATGAAGCACAGCCGCG	

MjakRpb1PCRF1	CGTGTGCTTCCGTACTCCACC	
MjakRpb1SF2	CCCAAGCCGGCTATTCTCAAGCC	
MjakRpb1SF3	GATGATTGCTTGTGTCGGCC	
MjakRpb1SF4	CCGCATCGACGTGCGTGTCGG	
MjakRpb1DSR1		GCTTCCGTACTCCACCTTCCG
MjakRpb1GSR2		CGACCGAGCGCGATGAAGTG
‘Malawimonas californiana’		
47Rpb1SF1	GGAACGTGCAGCCCAAGATT	
47Rpb1SF2	CACATTGATGGAAACGTTCGAGA	
47Rpb1SF5	CTGCATCTCGATTACGGATAC	
47Rpb1SF6	CATGTTGCCCCATCGCGTT	
47Rpb1SF7	CACGCGTTTTCATGAATGCT	
47Rpb1SR1		CATGGAATGCGATATTGTGCCT
47Rpb1SR2		GGCAGAGATTGAGGAGATT
